# Temporal variations in female moose responses to roads and logging in the absence of wolves

**DOI:** 10.1002/ece3.10909

**Published:** 2024-02-01

**Authors:** Mireille Gagnon, Frédéric Lesmerises, Martin‐Hugues St‐Laurent

**Affiliations:** ^1^ Centre for Forest Research, Département de Biologie Chimie et Géographie, Université du Québec à Rimouski Rimouski Québec Canada; ^2^ Direction de la Gestion de la faune du Bas‐Saint‐Laurent Ministère de l'Environnement, de la Lutte Contre les Changements Climatiques Rimouski Québec Canada; ^3^ Département de Biologie, Chimie et Géographie Université du Québec à Rimouski Rimouski Québec Canada; ^4^ Centre for Forest Research & Centre for Northern Studies, Département de Biologie, Chimie et Géographie Université du Québec à Rimouski Rimouski Québec Canada

**Keywords:** *Alces alces americana*, biological periods, daily phases, habitat selection, space use

## Abstract

Animal movements, needed to acquire food resources, avoid predation risk, and find breeding partners, are influenced by annual and circadian cycles. Decisions related to movement reflect a quest to maximize benefits while limiting costs, especially in heterogeneous landscapes. Predation by wolves (*Canis lupus*) has been identified as the major driver of moose (*Alces alces*) habitat selection patterns, and linear features have been shown to increase wolf efficiency to travel, hunt, and kill prey. However, few studies have described moose behavioral response to roads and logging in Canada in the absence of wolves. We thus characterized temporal changes (i.e., day phases and biological periods) in eastern moose (*Alces alces americana*) habitat selection and space use patterns near a road network in a wolf‐free area located south of the St. Lawrence River (eastern Canada). We used telemetry data collected on 18 females between 2017 and 2019 to build resource selection functions and mixed linear regressions to explain variations in habitat selection patterns, home‐range size, and movement rates. Female moose selected forest stands providing forage when movement was not impeded by snow cover (i.e., spring/green‐up, summer/rearing, fall/rut) and stands offering protection against incidental predation during calving. In winter, home‐range size decreased with an increasing proportion of stands providing food and shelter against harsh weather, limiting the energetic costs associated with movement. Our results reaffirmed the year‐round aversive effect of roads, even in the absence of wolves, but the magnitude of this avoidance differed between day phases, being lower during the “dusk‐night‐dawn” phase, perhaps due to a lower level of human activity on and near roads. Female moose behavior in our study area was similar to what was observed in landscapes where moose and wolves cohabit, suggesting that the risk associated with humans, perceived as another type of predator, and with incidental predators (coyote *Canis latrans*, black bear *Ursus americanus*), equates that of wolf predation in heavily managed landscapes.

## Graphical abstract

We characterized temporal changes in moose habitat selection and space use patterns near a road network in a wolf‐free area located in eastern Canada. Our results reaffirmed the year‐round aversive effect of roads, even in the absence of wolves, but the magnitude of this avoidance differed between day phases, being lower during the “dusk‐night‐dawn” phase, perhaps due to a lower level of human activity on and near roads. We found that moose behavior was similar to what was observed in landscapes where moose and wolves cohabit, suggesting that the risk associated with humans, perceived as another type of predator, outweighs that of wolf predation in heavily managed landscapes.

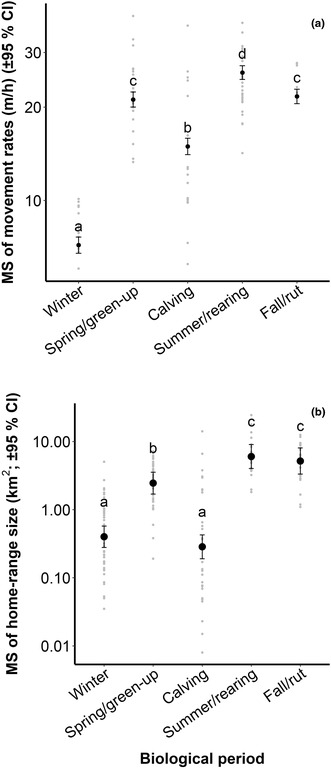

## 1 INTRODUCTION

Understanding the determinants of animal space use and habitat selection patterns implies taking into account circadian and annual cycles in animal behavior (e.g., Lagos et al., 2012; Stewart et al., 2022). Temporal variations can affect resource consumption (Merkle et al., 2016), predation (Kittle et al., 2022), and, consequently, individual survival (Jessop et al., 2018; Larsen & Boutin, 1994) and reproduction (Fahrig, 2007; Robertson et al., 2018). Merrow et al. (2005) described circadian cycles as a set of biochemical reactions modulated by temperature (Brown et al., 2002) and light (Spoelstra et al., 2004). This internal clock, common to every organism (Dibner & Schibler, 2015; Merrow et al., 2005), facilitates the anticipation of – and responses to – daily environmental changes (DeCoursey & Krulas, 1998; Rubin et al., 2017). By their influence on movements, these behavioral adjustments allow animals to optimize their food acquisition, predation avoidance, and search for breeding partners (Liedvogel et al., 2013; Nathan et al., 2008). In addition to circadian cycles, some energetic costs associated with movements (e.g., higher energy expenditure caused by snow cover: Bryce et al., 2022; Parker et al., 1984), quality and availability of food resources (Merkle et al., 2016), and the availability and necessity of shelters against predators and harsh weather conditions (Rueda et al., 2008) can vary between seasons. Individual space use and habitat selection patterns therefore reflect a quest to maximize benefits (e.g., territory marking: Wronski et al., 2006) and thereby limit costs (e.g., avoidance of biting insects: Vistnes et al., 2008; access to shelters against predation: Pokallus & Pauli, 2016) at different temporal scales (Baker & Rao, 2004; Larsen & Boutin, 1994).

In landscape ecology, the loss, alteration, and fragmentation of natural habitats can modulate the spatiotemporal distribution of resources and risks (Jaeger et al., 2005; Young et al., 2018). These habitat disturbances can originate from natural or anthropogenic causes (e.g., wildfires: Bosso et al., 2018; timber harvest: Hargis et al., 1999). Road networks also lead to these habitat disturbances (Cai et al., 2013; Fahrig et al., 2019) and can be considered as barriers to wildlife movement and dispersal (Haddad et al., 2015; Jaeger et al., 2005) depending on the species (Fahrig & Rytwinski, 2009) and road characteristics (Laurian et al., 2012; Leblond et al., 2011). Roads can trigger changes in animal behavior by altering movement patterns and escape responses (Trombulak & Frissell, 2000) or by acting as artificial home‐range boundaries (Mata et al., 2017). Animals usually perceive roads and traffic as a danger (Jaeger et al., 2005) and tend to space away from such linear features (Barocas et al., 2022; Polfus et al., 2011). When possible, they circumvent these barriers instead of crossing them (Ford & Fahrig, 2008; Shepard et al., 2008). Even though roads and traffic are mostly considered as stressors (Ditmer et al., 2018), there are situations where wildlife species could benefit from being close to roads (Fahrig & Rytwinski, 2009; Hill et al., 2021). For instance, roadsides can act as refuges for prey species through the aversive effect that traffic has on predators (Berger, 2007; Hebblewhite et al., 2005) or through fences that keep predators away (Ascensao et al., 2012), and can offer some reprieve to escape harassment from biting insects (Kelsall & Simpson, 1987). Roads can also provide an increased availability of food resources (e.g., access to young vegetation on roadsides in spring: Bowman et al., 2010) or important nutrients (e.g., minerals such as Na in roadside salt pools: Leblond et al., 2007), and facilitate movement (Dickie et al., 2017) as well as increase the encounter rate with prey for predators (Whittington et al., 2011).

According to Fahrig and Rytwinski (2009), large mammals with a low reproductive rate and a large home range are more likely to be negatively affected by road networks, in part because the larger the home ranges, the greater the probability of encountering roads (Jaeger et al., 2019). Roads are known to act as barriers to movement for many of these species, including caribou (*Rangifer tarandus caribou*: Dyer et al., 2002), red deer (*Cervus elaphu*s: Gagnon et al., 2007), and pronghorn (*Antilocapra americana*: Robb et al., 2022). Moose (*Alces alces*) is a long‐lived ungulate that produces 1–2 calves per year per female (Van Ballenberghe & Ballard, 2007), and its annual home‐range sizes vary from 13 to 130 km^2^ (Cederlund & Sand, 1994; Labonté et al., 1993). It is also the largest mammal commonly involved in wildlife – vehicle collisions in the northern hemisphere (Laliberté & St‐Laurent, 2020; Lavsund & Sandegren, 1991). Moose is thus an interesting biological model to disentangle the effects of landscape heterogeneity and anthropogenic disturbances on temporal variations in space use behavior and habitat selection patterns. Road networks are known to influence the movement patterns of moose, as they tend to move faster and over greater distances when close to paved (Wattles et al., 2018) and forest roads (Brown et al., 2018). These behavioral responses illustrate the compromises moose have to make to balance the risk of facing humans (Eldegard et al., 2012) or predators (DeMars & Boutin, 2018; St‐Pierre et al., 2022) near roads with the attractiveness of salt pools and early‐seral vegetation found on roadsides in spring (Laurian et al., 2008a; Miller & Litvaitis, 1992; Rea et al., 2021).

Moose behavioral responses vs. roads are well documented in areas where gray wolf (*Canis lupus*) is found (e.g., Quebec: Laurian et al., 2008a, 2008b, 2012; Ontario: Boyle et al., 2020; Scandinavia: Loosen et al., 2021), but fewer studies have focused on a landscape where this main predator has been extirpated (e.g., Norway: Eldegard et al., 2012). Our study thus aims at assessing, in the absence of wolf, the temporal variations in the space use and habitat selection patterns of female eastern moose (i.e., the North American subspecies, *Alces alces americana*) in a disturbed landscape characterized by a dense road network. Considering that behavior is known to vary between seasons, we defined 5 biological periods to address our objective: winter, spring/green‐up, calving, summer/rearing, and fall/rut. Also, since moose circadian cycles are in phase with luminosity (greater activity during dusk, night, and dawn; Haikonen & Summala, 2001; Krauze‐Gryz et al., 2017), we defined 2 day phases: day and dusk‐night‐dawn. Our hypothesis states that in the absence of wolf, female moose space use and habitat selection patterns will be less dependent on predation risk only, but instead driven by the trade‐off between resource acquisition, movement costs, and the mitigation of the risk of incidental predation by coyotes (*Canis latrans*) and black bears (*Ursus americanus*) on moose calves, with variations between day phases and biological periods (see Table S1 for an overview of our hypotheses and predictions). We thus predicted that female home ranges in winter will be (1) smaller due to higher energetic constraints associated with deep snow cover and (2) larger with a greater proportion of habitat that provides shelter against snow (e.g., mature mixed or coniferous forest stands). We also predicted that movement rates will be greater (3) during spring/green‐up, summer/rearing and fall/rut, as movements are not impeded by snow cover, (4) at dusk‐night‐dawn for all biological periods, as moose activity is known to be higher during these day phases, and (5) near paved and forest roads for all biological periods and day phases due to the compromise between the risk of facing humans or predators and the attractiveness of food resources located near roads (e.g., early seral vegetation found in regenerating stands). Finally, we predicted that female moose will select (6) habitats with greater availability of food resources (e.g., regenerating deciduous or mixed forest stands) at dusk‐night‐dawn during spring/green‐up, summer/rearing and fall/rut, (7) foraging opportunities found along paved and forest roads at dusk‐night‐dawn during spring/green‐up, summer/rearing and fall/rut, and 8) habitats offering shelter against incidental predation risk (e.g., taller coniferous forest stands) during calving, and they will stay closer to paved roads.

## 2 METHODS

### 2.1 Study area

**FIGURE 1:**
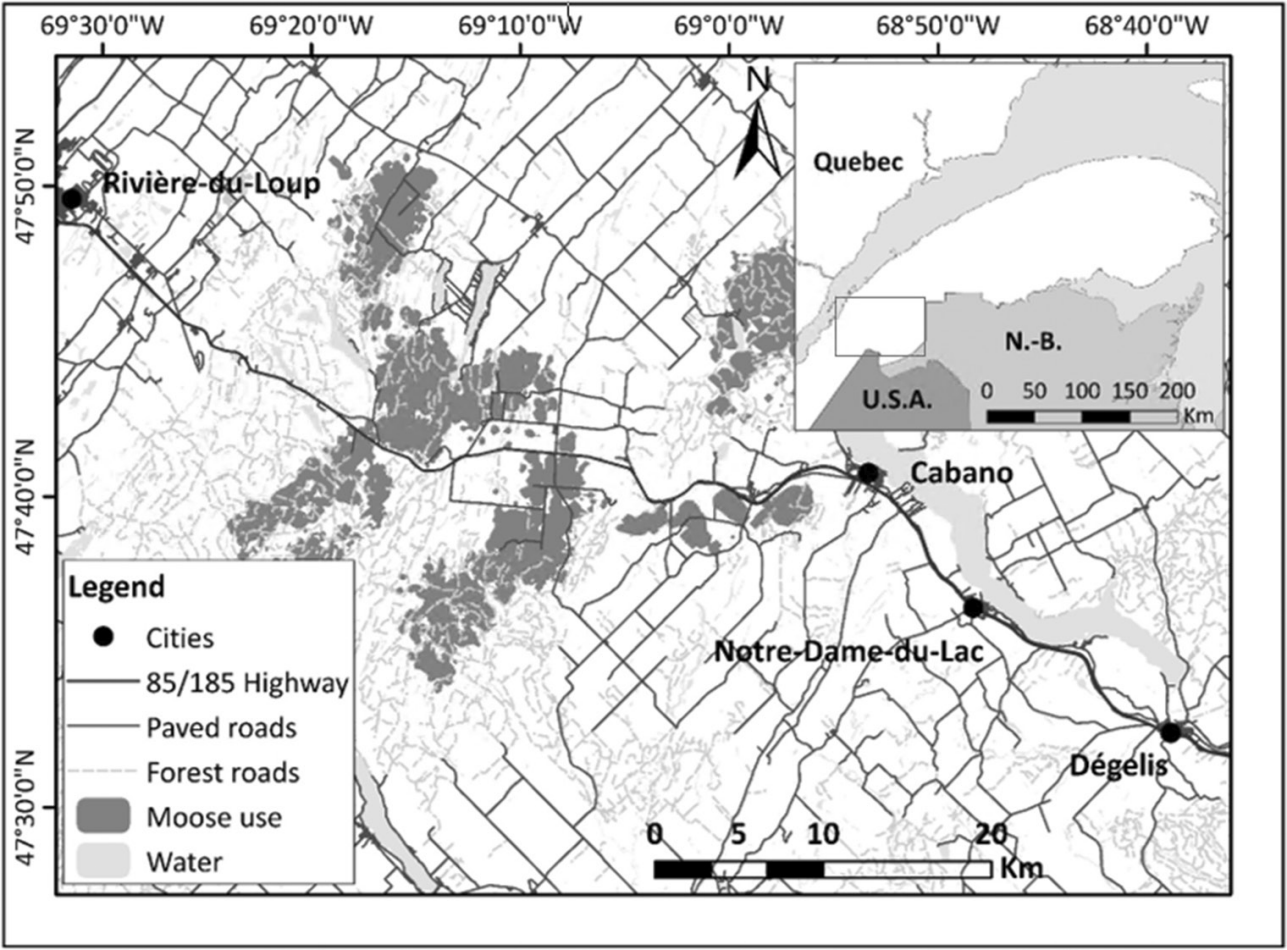
Study area near the Claude‐Béchard Highway (85–185) in the Bas‐Saint‐Laurent region, Canada. The area frequented by moose was estimated using the 95% kernel method (Brownian bridges: Horne et al., 2007; built with the *adehabitatHR* library: Calenge, 2021) with telemetry data collected on 18 female moose between 2017 and 2019.

The study took place in the Bas‐Saint‐Laurent region (Quebec, Canada; Figure 1), on the south shore of the St. Lawrence River, in the balsam fir (*Abies balsamea*) – yellow birch (*Betula alleghaniensis*) bioclimatic domain (Blouin & Berger, 2012). The study area encompasses the Claude‐Béchard Highway (85, formerly known as Highway 185) that connects the border of the province of New Brunswick to the city of Rivière‐du‐Loup (Quebec). The landscape is dominated by forest (~80%) and agriculture (~10%) with several small rural villages. This region has a subhumid, continental climate with mean annual temperatures of 2.5°C and is characterized by a mean elevation of 285 m with rolling hills (slopes of 7% on average; Robitaille & Saucier, 1998). Precipitation varies between 750 and 1250 mm, of which 35% is snow (Blouin & Berger, 2012). The vegetation is dominated by balsam fir, yellow birch, white birch (*Betula papyrifera*), white spruce (*Picea glauca*), and eastern white cedar (*Thuja occidentalis*) (Blouin & Berger, 2012). Moose, white‐tailed deer (*Odocoileus virginianus*), coyote, and black bear are the main large mammals observed in this region, and moose densities reach 5 moose/10 km^2^ of habitat (MFFP, 2016). The gray wolf was extirpated from the region ~170 years ago (Villemure & Jolicoeur, 2004). Moose densities are higher in this area than in regions where wolves are still found in the province of Quebec (e.g., 2.2/10 km^2^ in the Laurentides Wildlife Reserve, on the north shore of the St. Lawrence River; Rochette & Dumont, 2022). The high moose densities in our study area are also the result of intensive forest management practices deployed following the 1980–1990 spruce budworm (*Choristoneura fumiferana*) outbreak (Boulanger & Arseneault, 2004) and the ban on the use of chemical herbicides on Crown forest lands in Quebec since 2001 (Thompson & Pitt, 2003), which both provide abundant food resources to moose, as well as the implementation of sport hunting management strategies that promoted the growth of the population (Lefort & Massé, 2015). During data collection, the Claude‐Béchard Highway was under construction to upgrade the two‐lane road into a four‐lane highway. In addition to this 100 km‐long paved road, several forest and farm (gravel) roads (mean density = 1.04 km/km^2^) and small paved roads (mean density = 0.43 km/km^2^) crisscross the study area. The annual average daily traffic reaches ~7500 vehicles on Road 85/185 and ~1100 vehicles on smaller paved roads.

### 2.2 Capture and telemetry data

At the start of the project, we aimed to capture as many bull moose as cows but due to the very low abundance of bulls in the study area, we ended up capturing and collaring 2 males and 18 females that were monitored between 2017 and 2019 (for a total of 151,029 GPS locations). Capture and handling took place in February and March 2017, and our protocols were approved by the ministère de l'Environnement, de la Lutte contre les Changements Climatiques, de la Faune et des Parcs (wildlife management permit SEG # 2017‐02‐10‐010‐01‐S‐F) and by the Animal Welfare Committee of the Université du Québec à Rimouski (certificate CPA #68‐17‐183). We located moose by helicopter, and the selected individuals were directed to an open area where they could be darted to receive a dose of chemical immobilizer (9 mg of etorphine per animal). The dosage was adjusted for the sex and the mass estimated by the veterinarian who supervised and participated in the capture and handling operations. A GPS/Iridium telemetry collar (Vectronic Vertex Lite 3D) programmed to collect locations every 2 h was fitted to each animal's neck, and the antidote (270 mg of Naltraxone per animal) was administered before release. During capture and handling, 9 females were seen with a calf. Moose were monitored for 2.5 years after which the collars automatically fell off due to a drop‐off system. The location acquisition success of the collars was 99.2%. Among the 20 moose, 14 were followed for the entire or near‐complete monitoring, while 6 moose (2 males and 4 females) died a few months after capture, in most cases due to harvest by sport hunters. We thus removed the 2 males from our dataset because of the lack of data and to remove any confounding variability associated with the sex (see below how we dealt with the data of the 4 females that died). The telemetry data of the first 3 days were removed from the dataset to prevent a potential bias caused by the stress of capture and handling. We also removed GPS locations with a positional dilution of precision (hereafter PDOP) >10 (representing 0.1% of the dataset) to limit potential bias. We ended up with an average number of GPS locations per combination of individual (ID) – biological period that was high enough to conduct our statistical analyses (>300 locations annually and >100 locations seasonally for the home‐range size analysis, and far above 40 locations seasonally for the habitat selection analysis, following Girard et al., 2002, 2006; see the opening of the Results section).

### 2.3 Determination of biological periods and day phases

We delineated 5 biological periods (winter, spring/green‐up, calving, summer/rearing, fall/rut) and 2 day phases (day, dusk‐night‐dawn) to consider the temporal variations in moose behavior associated with these factors. We determined the cut‐off dates of the biological periods by identifying breaks in the distribution of mean movement rates in the function of Julian days and using the available knowledge on moose ecology (Hundertmark, 2007; Leblond et al., 2010); we did so for each ID – year combination to avoid generating biased results due to inter‐individual variation in behavior (Rudolph & Drapeau, 2012). For each year, we used the average dates (see Table S2) of each biological period for the moose that did not exhibit clear breaks in their movement rates (i.e., 17% of ID – biological period – year).

We defined day phases using the official sunrise and sunset times (National Research Council Canada, 2021): the day was bounded by the 60‐min period following sunrise to the 60‐min period preceding sunset, and dusk‐night‐dawn was bounded by the 60‐min period preceding sunset to the 60‐min period following sunrise. However, we ended up combining the dusk, dawn, and night phases to limit the number of categories in our analyses due to our small sample size, and because moose behavioral responses toward major paved roads were shown to be quite similar between these 3 day phases of low luminosity in our study area (see Laliberté & St‐Laurent, 2020).

### 2.4 Geomatics and spatial analyses

We defined landcover types using 1: 20,000 ecoforestry maps published by the ministère des Ressources naturelles et des Forêts (hereafter MRNF) and combined information from two mapping exercises (4^th^ and 5^th^ decennial inventories) to create updated annual maps to account for anthropogenic disturbance and fit the GPS data collected from our collared female moose (from 2017 to 2019). The minimum mapping size of the map was 4 ha for productive forest stands and <2 ha for non‐forested polygons (e.g., water bodies and agricultural fields). Resolution for forest operations updated annually could be as small as 0.1 ha. We regrouped the map polygons into a total of 8 landcover types relevant to moose ecology based on stand cover, composition, height, age, disturbance, land types, and representativity (Table 1). We decided to combine stands <4 m high (i.e., forest cuts and natural disturbances, covering ~4% of the study area) with other anthropogenic activities (covering ~14%, of which 83.1% were agricultural fields) because of the similar early successional vegetation they support, and uncategorized polygons (that represented only 0.3%) because of their low availability in the study area. Several of these landcover types are associated with known limiting factors (see the hypotheses in Table 1). While 0–4 m regenerating stands are known to provide several foraging opportunities in our region (Desgagnés et al., 2022), intermediate‐sized stands (4–7 m) are associated with an intermediate level of food resources when dominated by deciduous or mixed tree species; taller stands (>7 m) provide fewer foraging opportunities considering the size of the trees (Dussault et al., 2006). Conifer stands >4 m have lower lateral cover due to the natural thinning of stems, thus limiting movement costs, especially in winter, as the canopy intercepts snowfall (Dussault et al., 2006; Hundertmark et al., 1990). They can also contribute to risk mitigation, as coniferous stands support less fruit‐bearing shrubs and small prey attractive to bears and coyotes respectively (Boisjoly et al., 2010; Mosnier et al., 2008). We created a digital elevation model from 1: 20,000 hypsometry maps published by the MRNF to obtain topographic information, from which we calculated the slope (°) and the elevation (m). Both rasters had a 10 m × 10 m resolution. We classified roads into two categories using the 1: 20,000 Routard maps provided by the MRNF: (1) paved roads (e.g., Highway 85: average width of 107 m; road 185 and smaller local roads: average width of 34 m), and (2) forest roads (e.g., unpaved roads: average width of ~7 m), considering that the risk of human encounter (i.e., traffic vs. hunters) and the level of use by predators vary between these two road types.

**TABLE 1:** Description of each landcover type based on 1: 20,000 ecoforestry maps from the MRNF and its availability (%) in the area.

Landcover types	Description	Hypothesis	Availability (%)
0–4 m habitats	[0–4[m high stands, all cover types including habitats disturbed by anthropogenic activities (e.g., agriculture and habitation) and all the uncategorized polygons	Resource acquisition	17.7
4–7 m coniferous	[4–7[m high coniferous stands	Risk mitigation	1.8
4–7 m deciduous and mixed	[4–7[m high deciduous and mixed stands	Resource acquisition, Movement costs	2.4
7–12 m deciduous and mixed	[7–12[m high deciduous and mixed stands	–	5.6
+12 m deciduous and mixed	[+12[ m high deciduous and mixed stands	–	41.2
+7 m low‐density coniferous	[+7[ m high coniferous stands with 25%–60% density, including high‐density polygons that have been commercially thinned or partially cut	Movement costs, Risk mitigation	3.9
+7 m high‐density coniferous	[+7[ m high coniferous stands with 60% and over of density, excluding polygons that have been commercially thinned or partially cut	Movement costs, Risk mitigation	14.6
Wetlands and water bodies	Bogs, fens, marshes, lakes and rivers	–	12.8

#### 2.4.1 Space use patterns

We used moose movement rate and home‐range size to explain changes in space use patterns using habitat covariates while considering variations associated with biological periods and day phases (see Table S1). We estimated movement rates (in m/h) for each individual and each step (i.e., the trajectory linking two successive locations spaced by a 2 h interval) using Euclidean distances. Irregular time steps (6700 s > dt > 7700 s) were removed from the dataset for analyses. To contextualize the immediate surroundings of a step, we generated ellipses around each step using Brownian bridges (Horne et al., 2007) built with the *adehabitatHR* library (version 0.4.19; Calenge, 2021) in R 4.1.1 (R Core Team, 2021), as Lesmerises et al. (2015) did for black bears. We set two parameters (sig_1_ and sig_2_) a priori to calculate the Brownian bridges (Horne et al., 2007); sig_1_ referred to animal speed and was calculated with the liker function (Horne et al., 2007) for each ID – biological period (mean = 1.54, min = 0.22, max = 3.37), while sig_2_ referred to the standard deviation of the distance between GPS locations and real animal locations and was set at 5 considering that we already removed locations with a PDOP >10. Sensitivity tests with different sig_2_ (2, 5, 8, 10) also showed relatively small changes in the size of ellipses (mean variation of ±0.6%). We used the *getverticeshr* function with a probability of use >75% to delineate the ellipses to analyze movement rates at a fine scale while considering non‐rectilinear paths for moose. We calculated the proportion of landcover types, mean elevation (km), and slope (°) within the ellipses as well as coefficients of variation of slope. To test the importance of roads in explaining variation in moose movement rates, we calculated, for each GPS location, the minimum Euclidian distances to the nearest paved and forest road in ArcGIS 10.6.1 (ESRI, 2019).

We delineated seasonal home ranges using the kernel method based on Brownian bridges (Horne et al., 2007) with the *adehabitatHR* library (version 0.4.19; Calenge, 2021), using the same two parameters as for the ellipses (sig_1_ and sig_2_) with a probability of use >95% to consider animal movement. Under each of the 175 home ranges obtained (combinations of moose ID, biological periods, and years), we extracted the proportion of the different landcover types, the mean elevation (km), the mean slope (°), and the density of the two road classes (i.e., forest roads and all road types combined because of the low density of paved roads in the study area).

#### 2.4.2 Habitat selection patterns

We characterized female moose habitat selection patterns using resource selection functions (hereafter RSF; Manly et al., 2002) with the different landcover types and other covariates (elevation, slope, day phase, presence of forest and paved roads in a buffer zone) for each biological period. To do so, we delineated seasonal home ranges for each ID – biological period – year using 100% minimum convex polygons (MCP; Mohr, 1947) to adequately contrast use (moose locations) with availability (random points) (Laliberté & St‐Laurent, 2020; Leclerc et al., 2012). The number of random points distributed within the home ranges was equal to the number of GPS locations (i.e., a 1:1 ratio). For each moose location and random point, we extracted the landcover types as well as the elevation (km), slope (°), and minimum (Euclidian) distances (m) to the nearest paved and forest road using ArcGIS 10.6.1 (ESRI, 2019). Finally, we randomly attributed a day phase to the random points in the same proportion as what we had for the GPS locations for each ID – biological period.

### 2.5 Statistical analyses

For each biological period, we retained only combinations of ID – year for which we had data for the entire or nearly the entire duration of the biological period (i.e., ~4% of the ID – years were removed from the dataset for the statistical analyses). As our sample size was small, we aimed at limiting the number of covariates considered in the different regression models described below. To do so, we first conducted a priori tests with the different topography variables (slope, elevation, coefficient of variation of slope, alone or in combination) to retain only the variable (or combination of variables) found in the most parsimonious model for the subsequent analytical steps. These exploratory models based on topography variables were ranked using Akaike's Information Criterion corrected for small sample size (AIC_c_; Burnham & Anderson, 2001), and we repeated this step for each biological period. The topography variables retained for the space use pattern models changed according to the biological period for movement rates (i.e., elevation, slope, and coefficient of variation of slope in winter and spring/green‐up; elevation in calving and fall/rut; elevation and coefficient of variation of slope during summer/rearing) but was the same for home‐range size (i.e., elevation). For the habitat selection analyses, the slope and elevation were retained for winter, summer/rearing, and fall/rut, but the slope only was used for spring/green‐up and calving.

We had to convert the Euclidian distances to the nearest road into a dummy (binary) variable (i.e., the presence or absence of a road in a buffer zone surrounding each movement ellipse, for the movement rate analysis, and each GPS location or random point for the habitat selection analysis) to facilitate model convergence (as previously done by Laurian et al., 2012). We considered a range of buffer zone widths (0–75 m, 0–100 m, 0–150 m, 0–250 m, and 0–400 m) and ran our exploratory regression models using only the dummy variable (presence or absence of a road in the buffer zone) to rank the 5 different buffer zone widths based on the AIC_c_ for forest roads (based on Lesmerises et al., 2018). We repeated this approach for each biological period, as well as for paved roads. The buffer zone widths retained in the best‐ranked models for the movement rate regressions differed between forest roads (i.e., 0–75 m for all periods) and paved roads (i.e., 0–400 m in winter, 0–150 m in spring/green‐up, 0–100 m in calving, summer/rearing and fall/rut).

We repeated this procedure for the habitat selection analyses, and again the best‐ranked buffer zone widths differed between forest roads. The buffer size that proved to be the most parsimonious in our habitat selection models (based on AIC_c_) differed between road types and biological periods (forest roads = 0–75 m for all periods except fall/rut, during which it reached 0–100 m; paved roads = 0–150 m in winter and spring/green‐up, and 0–100 m in calving, summer/rearing and fall/rut). We used these buffer zone widths in the subsequent analyses.

We considered ID – year as a random factor in all the models described below to limit pseudo‐replication (Gillies et al., 2006) and consider the individual variability in behavioral responses (Duchesne et al., 2010), except for the analysis using home ranges, for which we used the ID. We built the linear regression models with the *lmerTest* library (version 3.1‐3; Kuznetsova et al., 2017). All statistical analyses were performed using R 4.1.1 (R Core Team, 2021).

#### 2.5.1 Space use patterns

We compared moose space use patterns between biological periods using an analysis of variance (ANOVA) with repeated measures followed by a multiple comparison test (Tukey) (Quinn & Keough, 2002). We log‐transformed movement rates and home‐range sizes (natural logarithm, *ln*) to meet the normality assumptions of this analysis.

We used two sets of linear mixed models to identify which variables explained the variation of movement rate and, in the second set, of home‐range size and repeated this for each biological period. The independent variables were the day phases (only for movement rates), the proportion of landcover type, the topography variables (see above), the presence of forest and paved roads in the buffer zone (see above; for movement rates), and the road density (for home‐range sizes). We built candidate models that represent our a priori hypotheses and predictions (see Table S1), ranked these models using AIC_c_ (see Tables S3 and S4), and estimated model adjustment using the pseudo‐*R* ^2^ (Zuur et al., 2007). We confirmed the normality of the residuals of these linear mixed models visually (Quinn & Keough, 2002). These linear regression models were run using the *lmerTest* library (version 3.1‐3; Kuznetsova et al., 2017).

#### 2.5.2 Habitat selection patterns

The RSF we used to describe the habitat selection patterns was a mixed logistic regression contrasting GPS locations (coded 1) with random points (coded 0) with different combinations of the following independent variables: landcover types, topography variables, day phases, and the presence of forest and paved roads in the buffer zone around each location (see above). We determined the reference habitat category by calculating the use/availability (U/A) ratio of each landcover type for each biological period. The category with the U/A ratio closest to 1 was the deciduous and mixed stands of +12 m in height for all biological periods (U/A ratio of 0.89 in winter, 1.15 in spring/green‐up, 1.08 during calving, 0.91 in summer/rearing, and 0.75 during fall/rut). We assessed multicollinearity between independent variables using the variance inflation factor (VIF) and found no issues associated with multicollinearity for our candidate models (i.e., VIF < 4) (Zuur et al., 2007). The candidate models (representing a priori hypotheses; see Tables S1 and S4) were ranked using AIC_c_, and we assessed the robustness of the most parsimonious model for each biological period using a k‐fold cross‐validation (Boyce et al., 2002; Johnson et al., 2006). To do so, we calculated parameter estimates by randomly selecting 75% of the observations and applied the resulting model to predict the values of the remaining 25%, and then calculated the Spearman‐rank correlation coefficient (*r*_*s*_; Zuur et al., 2007) by ranking the predicted values into 10 equal bins and comparing them with the frequency of real points in each bin. We repeated this procedure 39 times to obtain the mean *r*_*s*_ and its standard deviation. We ran the logistic regression models using the *lme4* library (version 1.1–27.1; Bates et al., 2015).

## 3 RESULTS

The telemetry monitoring conducted on 18 female moose yielded a variable – but sufficient – number of GPS locations per moose for all biological periods (i.e., winter: 3141 ± 1488 (SD); spring/green‐up: 598 ± 259; calving: 374 ± 161; summer/rearing: 2516 ± 587; fall/rut: 1291 ± 288).

### 3.1 Space use patterns

**FIGURE 2:**
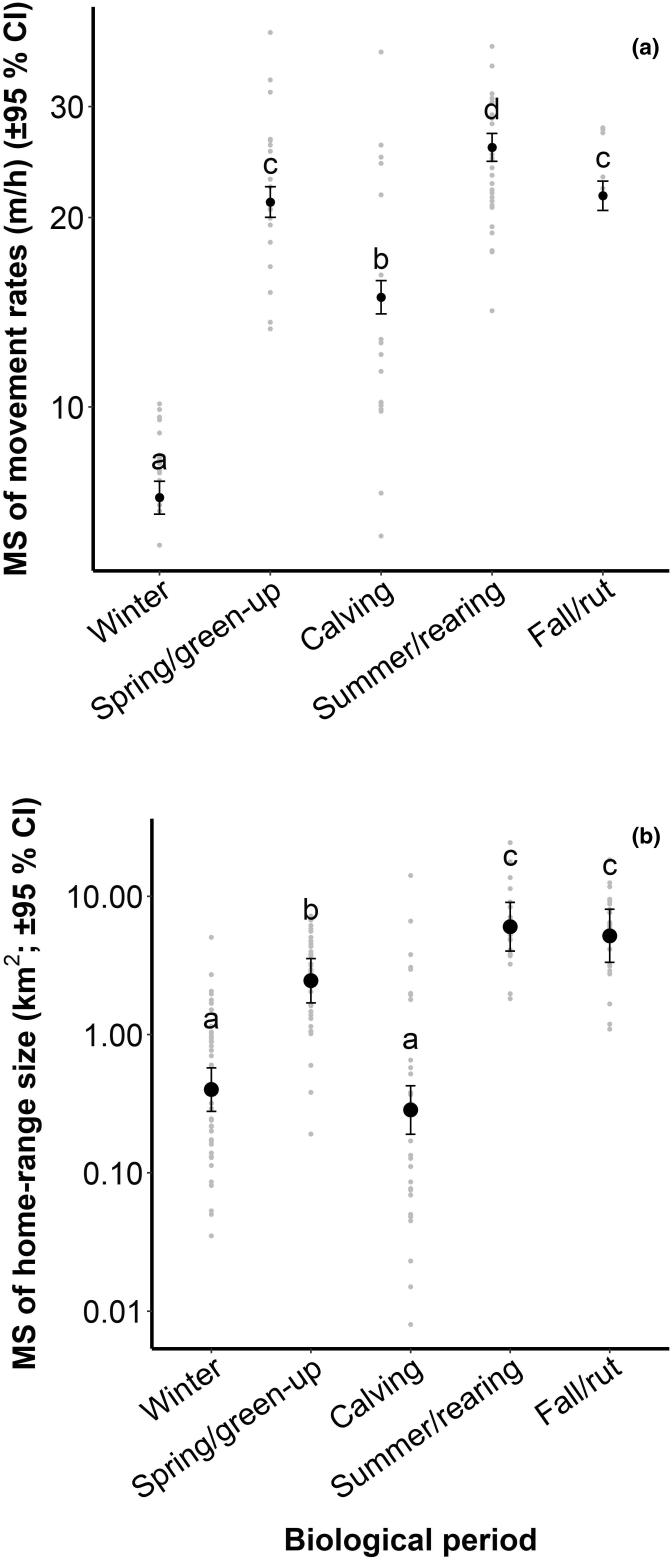
Mean squares (MS) of female moose movement rates (±95% CI) (a) and home‐range size (±95% CI) (b) according to the biological periods between 2017 and 2019 near the Claude‐Béchard Highway in the Bas‐Saint‐Laurent region, Canada. Mean square values with the same letter are not significantly different from each other. Data were log‐transformed (natural logarithm, *ln*).

**FIGURE 3:**
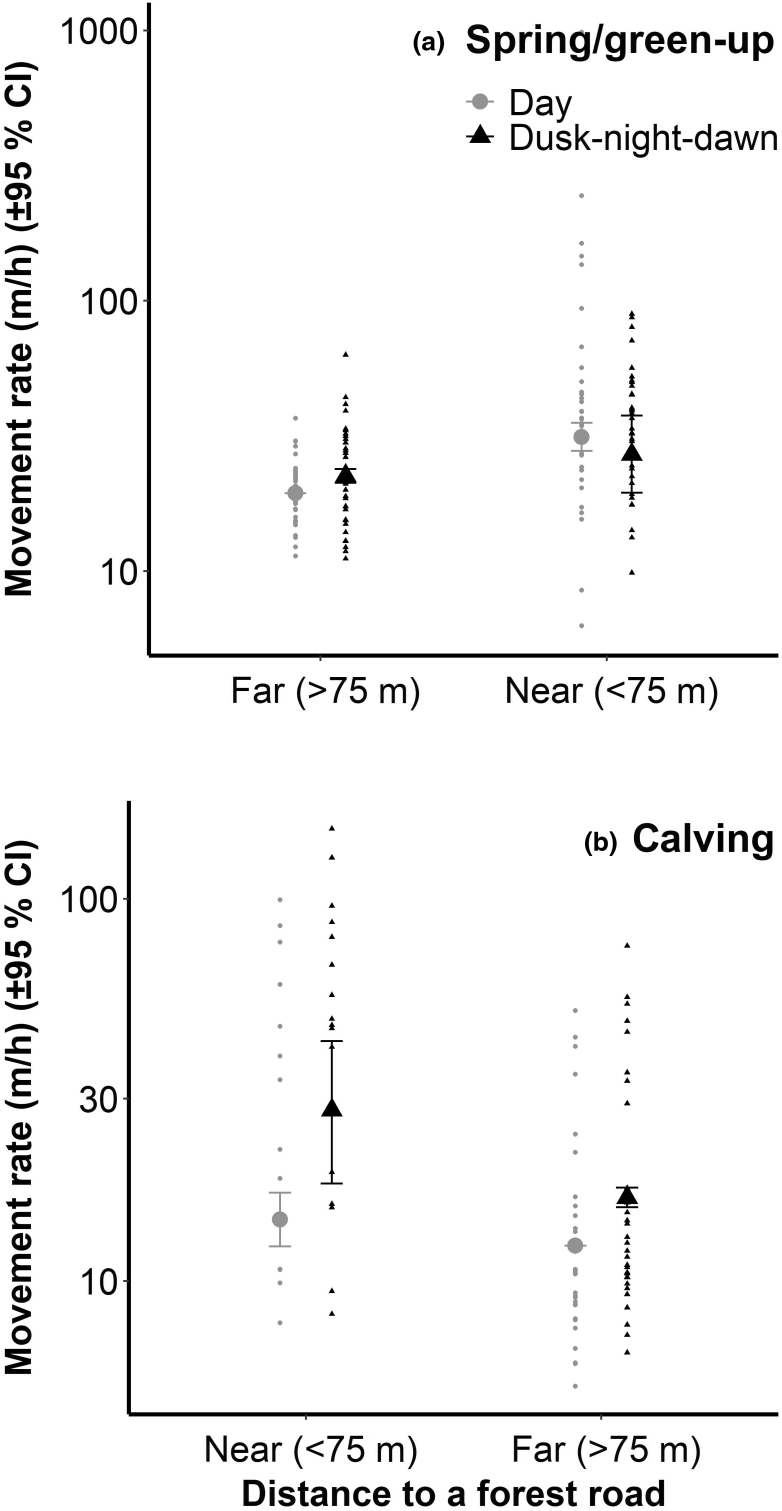
Predicted movement rate of female moose (±95% CI) and the interaction effect between the day phase and distance to a forest road during the spring/green‐up (a) and calving (b) biological periods between 2017 and 2019 near the Claude‐Béchard Highway in the Bas‐Saint‐Laurent region, Canada. Gray circles are for the “day” phase, and black triangles are for the “dusk‐night‐dawn” phase. Data were log‐transformed (natural logarithm, *ln*).

The ANOVA results showed that movement rates of female moose differed between biological periods (*F*_4,13_ = 5175.9; *p* < .001): all combinations of mean movement rates were significantly different (*p* < .001) except for spring/green‐up and fall/rut. Mean movement rates were higher in the summer/rearing biological period (25.84 m/h ± 0.67 SE), in fall/rut (21.65 m/h ± 0.60 SE), and in spring/green‐up (21.16 m/h ± 0.60 SE) (Figure 2a), supporting our third prediction. The most parsimonious mixed regression models explaining variations in movement rates for each biological period had a poor fit to the data (pseudo‐*R* ^2^ = .04 winter, 0.05 spring/green‐up, 0.28 calving, 0.08 summer/rearing, 0.05 fall/rut). Most of the variables had a significant effect on variations in movement rates. With regards to our fourth and fifth predictions, we noted that for summer/rearing, fall/rut, and winter, mean movement rates were higher during the “dusk‐night‐dawn” phase and near paved and forest roads (Table 2). The interaction between forest roads and day phases was also significant for the spring/green‐up and calving biological periods (Table 2), as mean movement rates were higher near forest roads (<75 m) during the “day” phase in spring/green‐up (Figure 3a). During calving, the mean movement rate was lower during the “day” phase whether moose were near (<75 m) or far (>75 m) from forest roads (Figure 3b).

**TABLE 2:** Coefficients estimates (*β*) and 95% confidence interval (95% CI; [Lower: Upper]) of the most parsimonious mixed regression model explaining variations in movement rates (m/h) of female moose for each biological period between 2017 and 2019 near the Claude‐Béchard Highway in the Bas‐Saint‐Laurent region, Canada. Coefficients for which the 95% CI did not overlap zero had a significant effect on the movement rates and are shown in bold. The day phase was used as the reference category.

Variables	Winter		Spring/green‐up		Calving		Summer/rearing		Fall/rut	
	*β*	95% CI	*β*	95% CI	*β*	95% CI	*β*	95% CI	*β*	95% CI
Intercept	**1.70**	**[1.57: 1.84]**	**3.20**	**[2.94: 3.48]**	**3.19**	**[2.71: 3.68]**	**2.58**	**[2.44: 2.71]**	**3.06**	**[2.83: 3.28]**
Elevation	**0.40**	**[0.07: 0.71]**	**−0.87**	**[−1.52: −0.20]**	**−1.95**	**[−3.08: −0.75]**	**0.88**	**[0.62: 1.18]**	**−0.60**	**[−1.07: −0.08]**
CV slope	**0.05**	**[0.04: 0.06]**	**0.06**	**[0.03: 0.09]**	–	–	**0.02**	**[0.01: 0.03]**	–	–
Slope	**0.06**	**[0.05: 0.07]**	**0.05**	**[0.02: 0.08]**	–	–	–	–	–	–
Dusk‐night‐dawn	**0.14**	**[0.12: 0.16]**	**0.14**	**[0.08: 0.20]**	**0.30**	**[0.24: 0.36]**	**0.54**	**[0.51: 0.56]**	**0.41**	**[0.36: 0.46]**
Paved roads	**0.10**	**[0.06: 0.13]**	**0.36**	**[0.12: 0.58]**	–	–	**0.49**	**[0.35: 0.63]**	**0.27**	**[0.12: 0.42]**
Forest roads	**0.18**	**[0.14: 0.22]**	**0.49**	**[0.35: 0.62]**	**0.18**	**[0.02: 0.35]**	**0.21**	**[0.16: 0.24]**	**0.08**	**[0.01: 0.14]**
0–4 m habitats	**0.29**	**[0.22: 0.36]**	–	–	–	–	−0.03	[−0.11: 0.04]	**−0.27**	**[−0.39: −0.15]**
4–7 m deciduous and mixed	**−0.13**	**[−0.18: −0.08]**	–	–	–	–	**−0.25**	**[−0.34: −0.17]**	**−0.30**	**[−0.43: −0.16]**
Dusk‐night‐dawn*Paved roads	–	–	−0.05	[−0.36: 0.28]	–	–	–	–	–	–
Dusk‐night‐dawn*Forest roads	–	–	**−0.30**	**[−0.46: −0.13]**	**0.34**	**[0.11: 0.57]**	–	–	–	–
Model fit (conditional *R* ^2^)	0.04		0.05		0.28		0.08		0.05	
Model fit (marginal *R* ^2^)	0.01		0.01		0.03		0.05		0.02	

The mean home‐range size of female moose differed between biological periods (*F*_4,155_ = 61.73; *p* < .001), with smaller home ranges during calving (0.29 km^2^ ± 0.06 SE) and winter (0.40 km^2^ ± 0.07 SE) and larger ones during summer/rearing (6.03 km^2^ ± 1.23 SE) and fall/rut (5.18 km^2^ ± 1.15 SE) (Figure 2b), thus partially supporting our first prediction. No difference in mean home‐range size was noted between winter and calving nor between summer/rearing and fall/rut. The most parsimonious models explaining home‐range size variations had a relatively good fit depending on the pseudo‐*R* ^2^ (conditional = 0.40 in winter, 0.88 for spring/green‐up, 0.91 during calving, 0.88 in summer/rearing, and 0.76 for fall/rut; marginal = 0.40 in winter, 0.10 for spring/green‐up, 0.43 during calving, 0.17 in summer/rearing, 0.08 for fall/rut). However, none of the fixed variables had a significant effect on variation in home‐range size except for the winter and calving periods (Table 3). An increase in home‐range size was associated with a greater proportion of 0–4 m (height) habitats and a higher road density in winter, and with a lower proportion of 4–7 m deciduous and mixed stands (Table 3), in opposition to our second prediction. During calving, the home‐range size increased with a greater density of forest roads and with an increasing proportion of 0–4 m habitats, although this latter effect was only marginally significant (i.e., the 95% confidence interval was strongly asymmetrical around zero; Table 3). A similar, marginally significant effect was noted during summer/rearing; an increasing proportion of +7 m low‐density coniferous stands was associated with a lower home‐range size.

**TABLE 3:** Coefficients estimates (*β*) and 95% confidence interval (95% CI; [Lower: Upper]) of the most parsimonious mixed regression model explaining variations in the home‐range size of female moose between 2017 and 2019 near the Claude‐Béchard Highway in the Bas‐Saint‐Laurent region, Canada. Coefficients for which the 95% CI did not overlap zero had a significant effect on the home‐range size and are shown in bold.

Variables	Winter		Spring/green‐up		Calving		Summer/rearing		Fall/rut	
	*β*	95% CI	*β*	95% CI	*β*	95% CI	*β*	95% CI	*β*	95% CI
Intercept	−0.73	[−3.05: 1.45]	0.22	[−1.68: 2.26]	−2.67	[−5.50: 0.27]	**3.34**	**[1.29: 5.42]**	1.28	[−2.18: 4.88]
Elevation	−0.55	[−5.93: 4.93]	0.08	[−4.56: 4.67]	0.82	[−7.25: 8.49]	−2.51	[−7.15: 2.51]	1.20	[−6.71: 9.08]
+7 m high‐density coniferous	−1.97	[−4.17: 0.71]	1.03	[−2.21: 4.19]	–	–	−1.57	[−5.11: 1.84]	−0.98	[−5.40: 3.27]
+7 m low‐density coniferous	−0.50	[−3.26: 2.42]	6.54	[−0.46: 13.16]	–	–	−5.31	[−11.02: 0.21]	−1.82	[−11.11: 7.09]
4–7 m deciduous and mixed	**−3.45**	**[−6.47: −0.31]**	1.39	[−2.28: 4.85]	2.04	[−1.33: 5.44]	−0.35	[−6.89: 5.66]	−0.26	[−10.74: 11.14]
0–4 m habitats	**8.62**	**[0.81: 16.94]**	1.98	[−1.25: 5.26]	5.22	[−0.45: 10.52]	1.72	[−5.27: 8.30]	2.45	[−3.05: 8.11]
Forest road density	–	–	–	–	**1.54**	**[0.88: 2.18]**	–	–	–	–
Road density	0.68	[0.23: 1.08]	–	–	–	–	–	–	–	–
Model fit (conditional *R* ^2^)	0.40		0.88		0.91		0.88		0.76	
Model fit (marginal *R* ^2^)	0.40		0.10		0.43		0.17		0.08	

### 3.2 Habitat selection patterns

**FIGURE 4:**
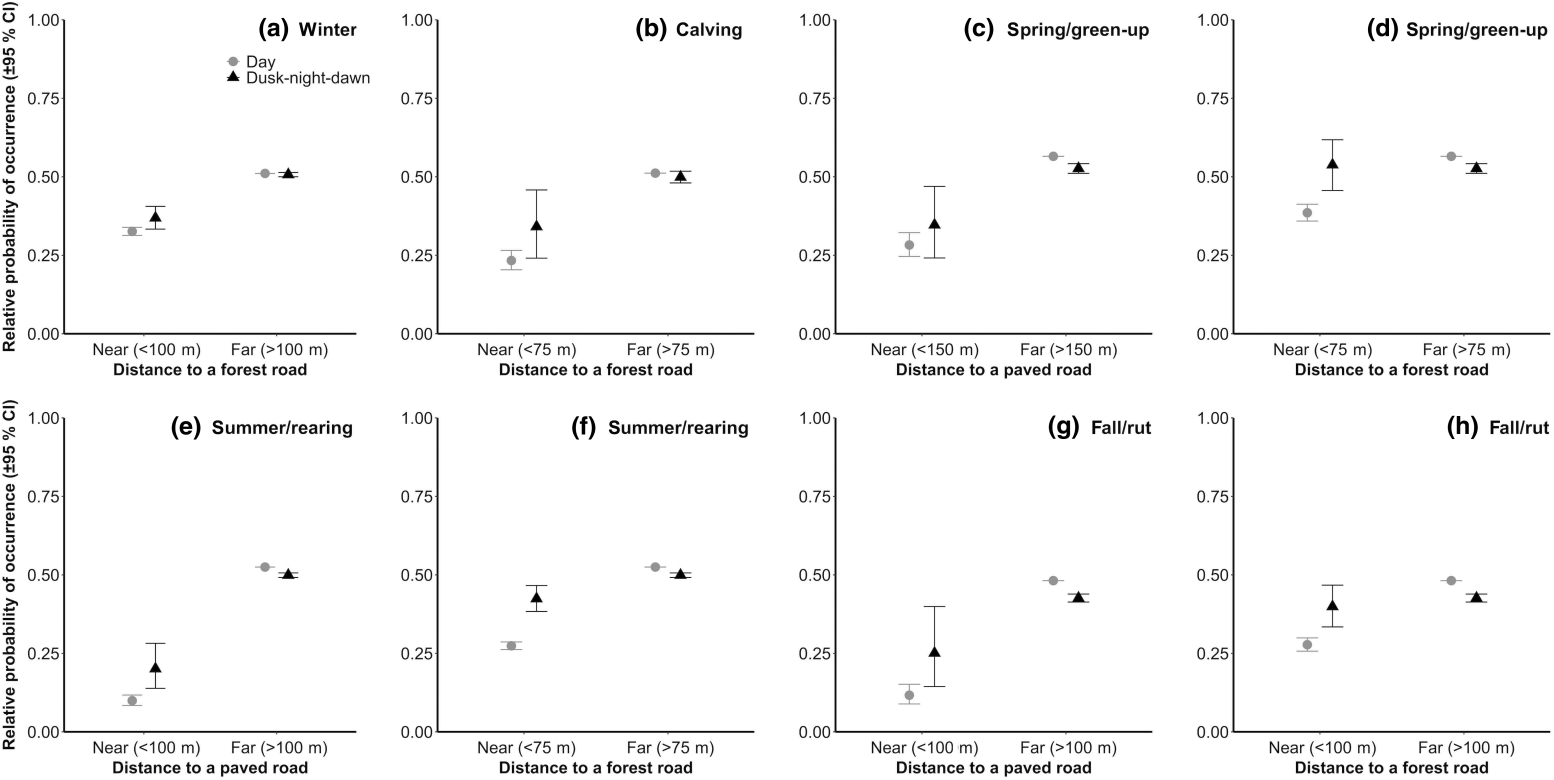
Interaction effect between day phase and distance to roads on the relative probability of female moose occurrence (±95% CI) for every biological period between 2017 and 2019 near the Claude‐Béchard Highway in the Bas‐Saint‐Laurent region, Canada: distance to a forest road for the winter (a) and calving (b) periods, distance to a paved road (c) and forest road (d) during spring/green‐up, distance to a paved road (e) and forest road (f) in summer/rearing and distance to a paved road (g) and forest road (h) during fall/rut. Gray circles are for the “day” phase, and black triangles are for the “dusk‐night‐dawn” phase.

The most parsimonious RSF models explaining variations in the probability of occurrence of female moose were robust to k‐fold cross‐validation for all biological periods (*r*_*s*_ = 0.89 ± 0.04 in winter, 0.97 ± 0.02 during spring/green‐up, 0.94 ± 0.03 during calving, 0.93 ± 0.04 in summer/rearing, 0.98 ± 0.01 in fall/rut). For spring/green‐up, summer/rearing, and fall/rut, moose selected 0–4 m (height) habitats and 4–7 m deciduous and mixed stands, and avoided +7 m coniferous stands that had a high density (Table 4), thus supporting our sixth prediction. During calving, females selected +7 m coniferous stands of both low and high densities but avoided 0–4 m habitats (Table 4). Similar trends were observed in winter; however, selection for coniferous stands tended toward zero (U/A ratio of the reference habitat category = 0.89). We found interactions between day phases and paved or forest roads for most biological periods (except for paved roads during winter and calving; Table 4), suggesting an increase in the relative probability of occurrence near (<75 m, 100 m, or 150 m depending on the biological period and road type) a forest or paved road during the “dusk‐night‐dawn” phase for all biological periods (Figure 4), providing only partial support to our seventh and eighth predictions.

**TABLE 4:** Coefficients estimates (*β*) and 95% confidence interval (95% CI; [Lower: Upper]) of the most parsimonious mixed logistic regression model explaining the relative probability of moose occurrence for each biological period between 2017 and 2019 near the Claude‐Béchard Highway in the Bas‐Saint‐Laurent region, Canada. Coefficients for which the 95% CI did not overlap zero had a significant effect on the relative probability of occurrence and are shown in bold. The deciduous and mixed stands +12 m and the day phase were used as the reference categories.

Variables	Winter		Spring/green‐up		Calving		Summer/rearing		Fall/rut	
	*β*	95% CI	*β*	95% CI	*β*	95% CI	*β*	95% CI	*β*	95% CI
Intercept	**2.34**	**[2.16: 2.53]**	**0.26**	**[0.21: 0.31]**	0.06	[−0.01: 0.13]	**−1.51**	**[−1.66: −1.36]**	**−1.18**	**[−1.41: −0.96]**
0–4 m habitats	**−0.28**	**[−0.35: −0.21]**	**0.37**	**[0.27: 0.47]**	**−0.62**	**[−0.82: −0.42]**	**0.34**	**[0.28: 0.39]**	**1.19**	**[1.11: 1.27]**
4–7 m coniferous	**0.09**	**[0.03: 0.16]**	**−0.15**	**[−0.29: −0.01]**	**−0.39**	**[−0.63: −0.14]**	−0.03	[−0.10: 0.04]	**0.90**	**[0.79: 1.01]**
4–7 m deciduous and mixed	0.02	[−0.04: 0.07]	**0.44**	**[0.34: 0.55]**	**0.66**	**[0.51: 0.80]**	**0.22**	**[0.16: 0.28]**	**1.13**	**[1.03: 1.22]**
7–12 m deciduous and mixed	**0.43**	**[0.39: 0.47]**	0.05	[−0.04: 0.14]	**0.19**	**[0.06: 0.31]**	**0.30**	**[0.25: 0.34]**	**0.54**	**[0.47: 0.61]**
+7 m high‐density coniferous	**0.05**	**[0.02: 0.09]**	**−0.83**	**[−0.93: −0.74]**	**0.12**	**[0.02: 0.22]**	**−0.04**	**[−0.08: <−0.01]**	**−0.16**	**[−0.23: −0.09]**
+7 m low‐density coniferous	**0.06**	**[0.01: 0.11]**	**−0.54**	**[−0.69: −0.40]**	**0.68**	**[0.51: 0.86]**	**0.26**	**[0.20: 0.32]**	**0.58**	**[0.48: 0.68]**
Wetlands and water bodies	**−1.40**	**[−1.50: −1.29]**	**−1.05**	**[−1.22: −0.89]**	−0.10	[−0.27: 0.07]	**−0.15**	**[−0.22: −0.09]**	−0.04	[−0.16: 0.08]
Elevation	**−5.79**	**[−6.19: −5.40]**	–	–	–	–	**4.04**	**[3.72: 4.35]**	**2.72**	**[2.20: 3.23]**
Slope	**−0.15**	**[−0.16: −0.14]**	**−0.04**	**[−0.07: −0.02]**	**0.14**	**[0.10: 0.19]**	**−0.09**	**[−0.10: −0.07]**	**−0.13**	**[−0.16: −0.11]**
Dusk‐night‐dawn	−0.01	[−0.04: 0.01]	**−0.16**	**[−0.22: −0.09]**	−0.05	[−0.13: 0.02]	**−0.10**	**[−0.13: −0.07]**	**−0.22**	**[−0.28: −0.17]**
Paved roads	**−0.40**	**[−0.52: −0.28]**	**−1.19**	**[−1.38: −1.01]**	**−1.16**	**[−1.47: −0.84]**	**−2.30**	**[−2.49: −2.12]**	**−1.95**	**[−2.25: −1.65]**
Forest roads	**−0.77**	**[−0.83: −0.71]**	**−0.73**	**[−0.84: −0.62]**	**−1.24**	**[−1.41: −1.06]**	**−1.07**	**[−1.13: −1.01]**	**−0.88**	**[−0.99: −0.78]**
Dusk‐night‐dawn*Paved roads	0.00	[−0.14: 0.15]	**0.45**	**[0.19: 0.72]**	0.38	[−0.06: 0.82]	**0.93**	**[0.70: 1.16]**	**1.15**	**[0.82: 1.49]**
Dusk‐night‐dawn*Forest roads	**0.20**	**[0.13: 0.27]**	**0.78**	**[0.62: 0.93]**	**0.58**	**[0.34: 0.83]**	**0.77**	**[0.69: 0.85]**	**0.77**	**[0.65: 0.89]**
Model fit (*r*_*s*_ by row ± SD)	0.89 ± 0.04		0.97 ± 0.02		0.94 ± 0.03		0.93 ± 0.04		0.98 ± 0.01	

## 4 DISCUSSION

Our results emphasized the variations in female moose behavior between day phases and biological periods and supported our hypothesis: in the absence of wolves, the behavior of female moose appears to be shaped by food resource acquisition and movement costs but also by the mitigation of predation risk by coyotes, bears, and humans. However, while our habitat selection models were robust and explained much of the observed variance, our capacity to highlight clear relationships between landscape covariates and movement rates or home‐range sizes was more limited.

### 4.1 Constraints associated with winter

As predicted, female moose selected food‐rich habitats and showed higher movement rates in most biological periods, except in winter, during which snow is known to impede movement (Dussault, Ouellet, et al., 2005), and during calving, when newborn calves have limited traveling capacities (Ballard & Van Ballenberghe, 2007). Other studies found similar results (higher movement rates during warm seasons in Quebec, see Leblond et al., 2010; a preference for food‐rich habitats, i.e., young and naturally regenerating stands in British Columbia, see Mumma et al., 2021). We showed that home ranges were smaller in winter, a possible consequence of greater energetic costs associated with movement in deep snow (Parker et al., 1984), as noted by Dussault, Courtois, et al. (2005) on the north shore of the St. Lawrence River. We also observed a decrease in home‐range size with an increasing proportion of habitats offering simultaneously food and shelter (i.e., 4–7 m deciduous and mixed stands), suggesting that the adjacency between these resources helped moose reduce their movements, an interpretation supported by their known selection for habitats offering both cover and food (e.g., Norway: Bjørneraas et al., 2011; Quebec: Dussault et al., 2006; Laurian et al., 2012). In opposition to our prediction, home‐range size did not increase with a greater proportion of sheltered habitats (i.e., +7 m coniferous stands of both low and high densities), perhaps because these stands do not offer good forage opportunities (Severud et al., 2019). However, home‐range size did increase with the proportion of 0–4 m habitats. Such habitats provide little shelter against snow (Dussault et al., 2006), especially since the majority of this category consisted of agricultural fields.

The influence of the landscape covariates we considered in our analyses on the variation in home‐range size was very small: there was no significant effect and only a small portion of explained variance, most of the variation being linked to interindividual variability. This suggests that some covariates were missing from our models. For example, this variation could be related to the difference in behavior between pregnant and lactating females (higher energy requirements: Richard et al., 2017) or females that are accompanied or not by a 6‐to‐9‐month‐old calf (Bowyer et al., 2001); this information was not available throughout our telemetry monitoring nor for all collared individuals.

### 4.2 Behavioral responses of moose to roads

Our results suggest that female moose showed an aversion to paved and forest roads, with higher movement rates near roads, larger home‐range sizes when road density was higher (all roads in winter and forest roads during calving), and a strong avoidance of areas close to roads during all biological periods. These results corroborate findings made by others regarding the aversive effect of roads on moose (e.g., Quebec: Laurian et al., 2008b, 2012; Norway: Eldegard et al., 2012) and other ungulates (e.g., roe deer, *Capreolus capreolus*, in Europe: Passoni et al., 2021; caribou in Alberta: Dyer et al., 2002). Such a negative response can be linked to human presence (e.g., cars and logging or agricultural equipment: Van Langevelde et al., 2009; hunters: Neumann & Ericsson, 2018) and traffic volume (Eldegard et al., 2012). The aversion may also have been exacerbated because of construction on Road 85/185 during the study, as noted on another road (Highway 175) by Leblond et al. (2013) on caribou and Lesmerises et al. (2013) on wolves. For forest roads in particular, avoidance by moose is usually explained by the use of roads by wolves (Lesmerises et al., 2012; Muhly et al., 2019), but finding similar patterns in an area where wolves are absent suggests that predation risk can still be an issue in a system where incidental predators are using roads to facilitate the patrol of their home range (bears: see DeMars & Boutin, 2018, as well as St‐Pierre et al., 2022; coyotes: see Kolbe et al., 2007, and Chow‐Fraser et al., 2022).

The fact that moose avoidance of roads was lower during the “dusk‐night‐dawn” phase suggests, however, that human disturbance might be the major driver of moose behavioral response toward roads. The probability of facing a predator during the “dusk‐night‐dawn” phase is higher (peak activity for wolves: Bryce et al., 2022, and coyotes: Thornton et al., 2004), so if predator avoidance was guiding moose response to roads, we should expect the avoidance of roads to be greater during this period, which is contradicted by our observations. Such a lower level of road avoidance during the “dusk‐night‐dawn” phase is in line with our prediction and is also supported by studies conducted in landscapes where wolves are still present (e.g., greater use of salt pools along paved roads: Leblond et al., 2007; more crossing of paved and forest roads: Laurian et al., 2008b; more moose‐vehicle collisions: Kučas & Balčiauskas, 2020). According to the general framework proposed by Frid and Dill (2002), moose may thus perceive humans as an important predation risk or at least as a source of disturbance, which can be triggered by the proximity of housing (Lykkja et al., 2009), by traffic volume (Wattles et al., 2018) or by hunters (Ausilio et al., 2022). During these day phases, moose have access to early‐seral vegetation (Bowman et al., 2010) and minerals (e.g., sodium in roadside salt pools during spring: Leblond et al., 2007) on roadsides, with minimum exposure to human disturbance (Eldegard et al., 2012). Movement rates were also higher during the “dusk‐night‐dawn” phase, a result supported by several research teams who have reported higher activity for other cervid species during these day phases (white‐tailed deer: Haikonen & Summala, 2001; roe deer: Krauze‐Gryz et al., 2017). We also noted a greater variation in the response of females near roads during the “dusk‐night‐dawn” phase. This variation could be linked to the difference in traffic volume (e.g., the average daily traffic in our study area varied between ~1100 and ~7500 vehicles, depending on the paved road type), or to an interindividual difference based on female reproductive status, known to influence behavior (Bowyer et al., 2001; Dussault, Ouellet, et al., 2005).

### 4.3 Avoiding risk imposed by incidental predators

In support of our prediction, female moose selected habitats providing shelter against incidental predation during calving (i.e., +7 m coniferous stands); these stand types do not necessarily provide a dense lateral cover (Pinard et al., 2012) but are known to be seldom used by coyotes near our study area (in Gaspésie, see Boisjoly et al., 2010). Alternatively, these stands could also provide shelter from high temperature and intense solar radiation during the warm season (Dussault et al., 2004). In areas where moose cohabit with wolves, female moose and their calves also favor conifer forests that lower predation risk or predator efficiency (Alaska: Bowyer et al., 2001; Quebec: Dussault, Ouellet, et al., 2005; Finland: Melin et al., 2019). Although there have been no wolves in our study area for ~170 years, black bears and coyotes, known to predate moose neonates (Ballard & Van Ballenberghe, 2007; Patterson & Messier, 2003), are relatively abundant and certainly shape habitat selection patterns of moose. The risk mitigation behavior we observed may also be due to moose's innate response acquired during cohabitation with their predator, as suggested by Chamaillé‐Jammes et al. (2014) for black‐tailed deer (*Odocoilus hemionus sitkensis*) in Canada, or by Makin et al. (2019) for African ungulates. However, confirming this alternative hypothesis would require additional effort, data (e.g., tissue samples), and genetic analyses, but represents an interesting avenue of research.

### 4.4 Limitations

Our inference potential is limited by a relatively small sample size of female moose, constraining the number of covariates included in our models (Tipton et al., 2017) and lowering statistical power (Quinn & Keough, 2002). For example, we were not able to contrast behavioral responses to roads and landcover types between dusk, night, and dawn and were forced to combine these day phases into a single category (vs. “day”). We also regrouped forest cuts and natural disturbances with other anthropogenic activities (agriculture) in order to balance our degrees of freedom more efficiently. Moreover, it was unfortunately impossible to identify all females that gave birth and how long the calf survived, but we recognize that behavioral responses can be influenced by female reproductive status (Bowyer et al., 2001; Dussault, Ouellet, et al., 2005), temperature (Street et al., 2015), snow depth (Melin et al., 2023) as well as hunting activity in the fall (Neumann & Ericsson, 2018). Such information would have had the potential to improve the fit of our models for the different analyses we conducted, especially for those aiming at explaining the variance in movement rate and home‐range size. For these analyses, the poor fit of our models suggests that some important covariates were not included, or that the variables we used were poor proxies of the limiting factors we considered (i.e., resource acquisition, movement costs, and risk mitigation), or even estimated at an inadequate spatiotemporal scale. Nevertheless, we are confident that the results we obtained for the habitat selection analyses were robust, especially based on the average number of telemetry locations as well as the number of collared moose that we had (Girard et al., 2002, 2006) and the strength of the k‐fold cross‐validation. Finally, we cannot extrapolate our results to male moose: although some evidence suggests that home‐range size (Cederlund & Sand, 1994; Laurian et al., 2008b) does not differ between sexes, habitat selection patterns sometimes differ between males and females (Joly et al., 2016) but sometimes do not (Herfindal et al., 2009).

## 5 CONCLUSION

We highlighted relationships that may suggest that female moose adjust their space use and habitat selection patterns to cope with the trade‐off between resource acquisition, movement costs, and mitigation of predation risk and human disturbance in a heavily altered landscape, even in the absence of a wolf. Behavioral responses to a heterogeneous landscape were similar to what was observed in landscapes where moose and wolves cohabit, suggesting that other forms of predation may affect moose behavior in our study area. Perhaps moose perceive humans as a greater predation risk, considering that they are an important game species in our study area (Lefort & Massé, 2015) and that humans can be considered as a “super predator” (Darimont et al., 2015). As an alternative explanation, our results also suggest that incidental predators such as coyotes and bears could supersede specialized predators (in our case wolves) in shaping the behavioral responses of a prey species. As mentioned earlier, some variables not considered in our study could also have contributed to shape the behavioral responses we observed. Our study has the advantage of providing additional information on female moose behavior in the absence of gray wolf in Canada, which was poorly covered in the literature. It also supports the possibility that humans are increasingly perceived as a significant disturbance in heavily altered landscapes, an avenue that should receive greater attention in future research.

## AUTHOR CONTRIBUTIONS

**Mireille Gagnon:** Formal analysis (equal); investigation (equal); validation (equal); writing – original draft (lead). **Frédéric Lesmerises:** Conceptualization (equal); formal analysis (equal); investigation (equal); methodology (equal); supervision (equal); validation (equal); writing – original draft (supporting); writing – review and editing (equal). **Martin‐Hugues St‐Laurent:** Conceptualization (equal); data curation (lead); formal analysis (supporting); funding acquisition (lead); investigation (equal); methodology (equal); project administration (lead); resources (lead); supervision (equal); validation (supporting); visualization (supporting); writing – original draft (supporting); writing – review and editing (equal).

## CONFLICT OF INTEREST STATEMENT

The authors declare that there is no conflict of interest.

## Supporting information


Appendix S1

## Data Availability

Data are archived on DRYAD and will be made openly available after manuscript acceptance. They can be consulted at https://doi.org/10.5061/dryad.dfn2z357t.

## References

[ece310909-bib-0001] Ascensao, F. , Clevenger, A. P. , Grilo, C. , Filipe, J. , & Santos‐Reis, M. (2012). Highway verges as habitat providers for small mammals in agrosilvopastoral environments. Biodiversity and Conservation, 21, 3681–3697.

[ece310909-bib-0002] Ausilio, G. , Wikenros, C. , Sand, H. , Wabakken, P. , Eriksen, A. , & Zimmermann, B. (2022). Environmental and anthropogenic features mediate risk from human hunters and wolves for moose. Ecosphere, 13, e4323.

[ece310909-bib-0003] Baker, M. B. , & Rao, S. (2004). Incremental costs and benefits shape natal dispersal: Theory and example with *Hemilepistus reaumuri* . Ecology, 85, 1039–1051.

[ece310909-bib-0004] Ballard, W. B. , & Van Ballenberghe, V. (2007). Predator – Prey relationships. In C. C. Schwartz , A. E. Franzmann , & R. E. McCabe (Eds.), Ecology and Management of the North American Moose (2nd ed., pp. 247–274). University Press of Colorado.

[ece310909-bib-0005] Barocas, A. , Hefner, R. , Ucko, M. , Shalmon, B. , Leader, N. , & Geffen, E. (2022). Ruppell's fox movement and spatial behavior are influenced by topography and human activity. Biodiversity and Conservation, 31, 1345–1357.

[ece310909-bib-0006] Bates, D. , Mächler, M. , Bolker, B. , & Walker, S. (2015). Fitting linear mixed‐effects models using lme4. Journal of Statistical Software, 67, 1–48.

[ece310909-bib-0007] Berger, J. (2007). Fear, human shields and the redistribution of prey and predators in protected areas. Biology Letters, 3, 620–623. 17925272 10.1098/rsbl.2007.0415 PMC2391231

[ece310909-bib-0008] Bjørneraas, K. , Solberg, E. J. , Herfindal, I. , Moorter, B. V. , Rolandsen, C. M. , Tremblay, J.‐P. , Skarpe, C. , Sæther, B.‐E. , Eriksen, R. , & Astrup, R. (2011). Moose *Alces alces* habitat use at multiple temporal scales in a human‐altered landscape. Wildlife Biology, 17, 44–54.

[ece310909-bib-0009] Blouin, J. , & Berger, J. P. (2012). Guide de reconnaissance des types écologiques de la région écologique 4f – Collines des moyennes Appalaches (2nd ed.). Ministère des Ressources naturelles et de la Faune – Forêt Québec [French].

[ece310909-bib-0010] Boisjoly, D. , Ouellet, J.‐P. , & Courtois, R. (2010). Coyote habitat selection and management implications for the Gaspésie caribou. Journal of Wildlife Management, 74, 3–11.

[ece310909-bib-0011] Bosso, L. , Ancillotto, L. , Smeraldo, S. , D'Arco, S. , Migliozzi, A. , Conti, P. , & Russo, D. (2018). Loss of potential bat habitat following a severe wildfire: A model‐based rapid assessment. International Journal of Wildland Fire, 27, 756–769.

[ece310909-bib-0012] Boulanger, Y. , & Arseneault, D. (2004). Spruce budworm outbreaks in eastern Quebec over the last 450 years. Canadian Journal of Forest Research, 34, 1035–1043.

[ece310909-bib-0013] Bowman, J. , Ray, J. C. , Magoun, A. J. , Johnson, D. S. , & Dawson, F. N. (2010). Roads, logging, and the large‐mammal community of an eastern Canadian boreal forest. Canadian Journal of Zoology, 88, 454–467.

[ece310909-bib-0014] Bowyer, R. T. , Pierce, B. M. , Duffy, L. K. , & Haggstrom, D. A. (2001). Sexual segregation in moose: Effects of habitat manipulation. Alces, 37, 109–123.

[ece310909-bib-0015] Boyce, M. S. , Vernier, P. R. , Nielsen, S. E. , & Schmiegelow, F. K. A. (2002). Evaluating resource selection functions. Ecological Modelling, 157, 281–300.

[ece310909-bib-0016] Boyle, S. P. , Litzgus, J. D. , & Lesbarrères, D. (2020). Limited evidence for negative effects of highway widening on north American large mammals. European Journal of Wildlife Research, 66, 1–10.

[ece310909-bib-0017] Brown, C. L. , Kielland, K. , Brinkman, T. J. , Gilbert, S. L. , & Euskirchen, E. S. (2018). Resource selection and movement of male moose in response to varying levels of off‐road vehicle access. Ecosphere, 9, e02405.

[ece310909-bib-0018] Brown, S. A. , Zumbrunn, G. , Fleury‐Olela, F. , Preitner, N. , & Schibler, U. (2002). Rhythms of mammalian body temperature can sustain peripheral circadian clocks. Current Biology, 12, 1574–1583. 12372249 10.1016/s0960-9822(02)01145-4

[ece310909-bib-0019] Bryce, C. M. , Dunford, C. E. , Pagano, A. M. , Wang, Y. , Borg, B. L. , Arthur, S. M. , & Williams, T. M. (2022). Environmental correlates of activity and energetics in a wide‐ranging social carnivore. Animal Biotelemetry, 10, 1–16.

[ece310909-bib-0020] Burnham, K. P. , & Anderson, D. R. (2001). Kullback–Leibler information as a basis for strong inference in ecological studies. Wildlife Research, 28, 111–119.

[ece310909-bib-0021] Cai, X. , Wu, Z. , & Cheng, J. (2013). Using kernel density estimation to assess the spatial pattern of road density and its impact on landscape fragmentation. International Journal of Geographical Information Science, 27, 222–230.

[ece310909-bib-0022] Calenge, C. (2021). *Home range estimation in R: The adehabitatHR package*. The Comprehensive R Archive Network. https://mran.microsoft.com/snapshot/2016‐08‐05/web/packages/adehabitatHR/vignettes/adehabitatHR.pdf.

[ece310909-bib-0023] Cederlund, G. , & Sand, H. (1994). Home‐range size in relation to age and sex in moose. Journal of Mammalogy, 75, 1005–1012.

[ece310909-bib-0024] Chamaillé‐Jammes, S. , Malcuit, H. , Le Saout, S. , & Martin, J. L. (2014). Innate threat‐sensitive foraging: Black‐tailed deer remain more fearful of wolf than of the less dangerous black bear even after 100 years of wolf absence. Oecologia, 174, 1151–1158. 24288079 10.1007/s00442-013-2843-0

[ece310909-bib-0025] Chow‐Fraser, G. , Heim, N. , Paczkowski, J. , Volpe, J. P. , & Fisher, J. T. (2022). Landscape change shifts competitive dynamics between declining at‐risk wolverines and range‐expanding coyotes, compelling a new conservation focus. Biological Conservation, 266, 109435.

[ece310909-bib-0026] Darimont, C. T. , Fox, C. H. , Bryan, H. M. , & Reimchen, T. E. (2015). The unique ecology of human predators. Science, 349, 858–860. 26293961 10.1126/science.aac4249

[ece310909-bib-0027] DeCoursey, P. J. , & Krulas, J. R. (1998). Behavior of SCN‐lesioned chipmunks in natural habitat: A pilot study. Journal of Biological Rhythms, 13, 229–244. 9615287 10.1177/074873098129000075

[ece310909-bib-0028] DeMars, C. A. , & Boutin, S. (2018). Nowhere to hide: Effects of linear features on predator–prey dynamics in a large mammal system. Journal of Animal Ecology, 87, 274–284. 28940254 10.1111/1365-2656.12760

[ece310909-bib-0029] Desgagnés, J.‐F. , Schneider, R. , & St‐Laurent, M.‐H. (2022). Winter browsing in absence of an apical predator: Do high moose densities compromise tree regeneration? Forest Ecology and Management, 520, 120403.

[ece310909-bib-0030] Dibner, C. , & Schibler, U. (2015). Circadian timing of metabolism in animal models and humans. Journal of Internal Medicine, 277, 513–527. 25599827 10.1111/joim.12347

[ece310909-bib-0031] Dickie, M. , Serrouya, R. , McNay, R. S. , & Boutin, S. (2017). Faster and farther: Wolf movement on linear features and implications for hunting behaviour. Journal of Applied Ecology, 54, 253–263.

[ece310909-bib-0032] Ditmer, M. A. , Rettler, S. J. , Fieberg, J. R. , Iaizzo, P. A. , Laske, T. G. , Noyce, K. V. , & Garshelis, D. L. (2018). American black bears perceive the risks of crossing roads. Behavioral Ecology, 29, 667–675.

[ece310909-bib-0033] Duchesne, T. , Fortin, D. , & Courbin, N. (2010). Mixed conditional logistic regression for habitat selection studies. Journal of Animal Ecology, 79, 548–555. 20202010 10.1111/j.1365-2656.2010.01670.x

[ece310909-bib-0034] Dussault, C. , Courtois, R. , & Ouellet, J.‐P. (2006). A habitat suitability index model to assess moose habitat selection at multiple spatial scales. Canadian Journal of Forest Research, 36, 1097–1107.

[ece310909-bib-0035] Dussault, C. , Courtois, R. , Ouellet, J.‐P. , & Girard, I. (2005). Space use of moose in relation to food availability. Canadian Journal of Zoology, 83, 1431–1437.

[ece310909-bib-0036] Dussault, C. , Ouellet, J.‐P. , Courtois, R. , Huot, J. , Breton, L. , & Jolicoeur, H. (2005). Linking moose habitat selection to limiting factors. Ecography, 28, 619–628.

[ece310909-bib-0037] Dussault, C. , Ouellet, J.‐P. , Courtois, R. , Huot, J. , Breton, L. , & Larochelle, J. (2004). Behavioural responses of moose to thermal conditions in the boreal forest. Ecoscience, 11, 321–328.

[ece310909-bib-0038] Dyer, S. J. , O'Neill, J. P. , Wasel, S. M. , & Boutin, S. (2002). Quantifying barrier effects of roads and seismic lines on movements of female woodland caribou in northeastern Alberta. Canadian Journal of Zoology, 80, 839–845.

[ece310909-bib-0039] Eldegard, K. , Lyngved, J. T. , & Hjeljord, O. (2012). Coping in a human‐dominated landscape: Trade‐off between foraging and keeping away from roads by moose (*Alces alces*). European Journal of Wildlife Research, 58, 969–979.

[ece310909-bib-0040] Environmental Systems Research Institute (ESRI) . (2019). ArcGIS version 10.6.1. Environmental Systems Research Institute Inc.

[ece310909-bib-0041] Fahrig, L. (2007). Non‐optimal animal movement in human‐altered landscapes. Functional Ecology, 21, 1003–1015.

[ece310909-bib-0042] Fahrig, L. , Arroyo‐Rodríguez, V. , Bennett, J. R. , Boucher‐Lalonde, V. , Cazetta, E. , Currie, D. J. , Eigenbrod, F. , Ford, A. T. , Harrison, S. P. , Jaeger, J. A. G. , Koper, N. , Martin, A. E. , Martin, J. L. , Metzger, J. P. , Morrison, P. , Rhodes, J. R. , Saunders, D. A. , Simberloff, D. , Smith, A. C. , … Watling, J. I. (2019). Is habitat fragmentation bad for biodiversity? Biological Conservation, 230, 179–186.

[ece310909-bib-0043] Fahrig, L. , & Rytwinski, T. (2009). Effects of roads on animal abundance: An empirical review and synthesis. Ecology and Society, 14, 21–41.

[ece310909-bib-0044] Ford, A. T. , & Fahrig, L. (2008). Movement patterns of eastern chipmunks (*Tamias striatus*) near roads. Journal of Mammalogy, 89, 895–903.

[ece310909-bib-0045] Frid, A. , & Dill, L. (2002). Human‐caused disturbance stimuli as a form of predation risk. Conservation Ecology, 6, 11.

[ece310909-bib-0046] Gagnon, J. W. , Theimer, T. C. , Dodd, N. L. , Boe, S. , & Schweinsburg, R. E. (2007). Traffic volume alters elk distribution and highway crossings in Arizona. Journal of Wildlife Management, 71, 2318–2323.

[ece310909-bib-0047] Gillies, C. S. , Hebblewhite, M. , Nielsen, S. E. , Krawchuk, M. A. , Aldridge, C. L. , Frair, J. L. , Saher, D. J. , Stevens, M. C. E. , & Jerde, C. L. (2006). Application of random effects to the study of resource selection by animals. Journal of Animal Ecology, 75, 887–898. 17009752 10.1111/j.1365-2656.2006.01106.x

[ece310909-bib-0048] Girard, I. , Dussault, C. , Ouellet, J.‐P. , Courtois, R. , & Caron, A. (2006). Balancing number of locations with number of individuals in telemetry studies. Journal of Wildlife Management, 70, 1249–1256.

[ece310909-bib-0049] Girard, I. , Ouellet, J.‐P. , Courtois, R. , Dussault, C. , & Breton, L. (2002). Effects of sampling effort based on GPS telemetry on home‐range size estimations. Journal of Wildlife Management, 66, 1290–1300.

[ece310909-bib-0050] Haddad, N. M. , Brudvig, L. A. , Clobert, J. , Davies, K. F. , Gonzalez, A. , Holt, R. D. , Lovejoy, T. E. , Sexton, J. O. , Austion, M. P. , Collins, C. D. , Cook, W. M. , Damschen, E. I. , Ewers, R. M. , Foster, B. L. , Jenkins, C. N. , King, A. J. , Laurance, W. F. , Levey, D. J. , Margules, C. R. , … Townshend, J. R. (2015). Habitat fragmentation and its lasting impact on Earth's ecosystems. Science Advances, 1, 1–9. 10.1126/sciadv.1500052 PMC4643828 26601154

[ece310909-bib-0051] Haikonen, H. , & Summala, H. (2001). Deer‐vehicle crashes: Extensive peak at 1 hour after sunset. American Journal of Preventive Medicine, 21, 209–213. 11567842 10.1016/s0749-3797(01)00352-x

[ece310909-bib-0052] Hargis, C. D. , Bissonette, J. A. , & Turner, D. L. (1999). The influence of forest fragmentation and landscape pattern on American martens. Journal of Applied Ecology, 36, 157–172.

[ece310909-bib-0053] Hebblewhite, M. , White, C. A. , Nietvelt, C. G. , McKenzie, J. A. , Hurd, T. E. , Fryxell, J. M. , Bayley, S. E. , & Paquet, P. C. (2005). Human activity mediates a trophic cascade caused by wolves. Ecology, 86, 2135–2144.

[ece310909-bib-0054] Herfindal, I. , Tremblay, J.‐P. , Hansen, B. B. , Solberg, E. J. , Heim, M. , & Sæther, B.‐E. (2009). Scale dependency and functional response in moose habitat selection. Ecography, 32, 849–859.

[ece310909-bib-0055] Hill, J. E. , DeVault, T. L. , & Belant, J. L. (2021). A review of ecological factors promoting road use by mammals. Mammal Review, 51, 214–227.

[ece310909-bib-0056] Horne, J. S. , Garton, E. O. , Krone, S. M. , & Lewis, J. S. (2007). Analyzing animal movements using Brownian bridges. Ecology, 88, 2354–2363. 17918412 10.1890/06-0957.1

[ece310909-bib-0057] Hundertmark, K. J. (2007). Home range, dispersal and migration. In C. C. Schwartz , A. E. Franzmann , & R. E. McCabe (Eds.), Ecology and Management of the North American Moose (2nd ed., pp. 303–336). University Press of Colorado.

[ece310909-bib-0058] Hundertmark, K. J. , Eberhardt, W. L. , & Ball, R. E. (1990). Winter habitat use by moose in southeastern Alaska: Implications for forest management. Alces, 26, 108–114.

[ece310909-bib-0059] Jaeger, J. , Spanowicz, A. , Bowman, J. , & Clevenger, A. (2019). Clôtures et passages fauniques pour les petits et moyens mammifères le long de la route 175 au Québec: Quelle est leur efficacité? Naturaliste Canadien, 143, 69–80. [French].

[ece310909-bib-0060] Jaeger, J. A. , Bowman, J. , Brennan, J. , Fahrig, L. , Bert, D. , Bouchard, J. , Charbonneau, N. , Frank, K. , Gruber, B. , & Von Toschanowitz, K. T. (2005). Predicting when animal populations are at risk from roads: An interactive model of road avoidance behavior. Ecological Modelling, 185, 329–348.

[ece310909-bib-0061] Jessop, T. S. , Webb, J. , Dempster, T. , Feit, B. , & Letnic, M. (2018). Interactions between corticosterone phenotype, environmental stressor pervasiveness and irruptive movement‐related survival in the cane toad. Journal of Experimental Biology, 221, 187930. 10.1242/jeb.187930 30352824

[ece310909-bib-0062] Johnson, C. J. , Nielsen, S. E. , Merrill, E. H. , McDonald, T. L. , & Boyce, M. S. (2006). Resource selection functions based on use‐availability data: Theoretical motivation and evaluation methods. Journal of Wildlife Management, 70, 347–357.

[ece310909-bib-0063] Joly, K. , Sorum, M. S. , Craig, T. , & Julianus, E. L. (2016). The effects of sex, terrain, wildfire, winter severity, and maternal status on habitat selection by moose in north‐central Alaska. Alces, 52, 101–115.

[ece310909-bib-0064] Kelsall, J. P. , & Simpson, K. (1987). The impacts of highways on ungulates: A review and selected bibliography. BC Ministry of Environment and Parks.

[ece310909-bib-0065] Kittle, A. M. , Bukombe, J. K. , Sinclair, A. R. E. , Mduma, S. A. R. , & Fryxell, J. M. (2022). Where and when does the danger lie? Assessing how location, season and time of day affect the sequential stages of predation by lions in western Serengeti National Park. Journal of Zoology, 316, 229–239.

[ece310909-bib-0066] Kolbe, J. A. , Squires, J. R. , Pletscher, D. H. , & Ruggiero, L. F. (2007). The effect of snowmobile trails on coyote movements within lynx home ranges. Journal of Wildlife Management, 71, 1409–1418.

[ece310909-bib-0067] Krauze‐Gryz, D. , Żmihorski, M. , Jasińska, K. , Kwaśny, L. , & Werka, J. (2017). Temporal pattern of wildlife‐train collisions in Poland. Journal of Wildlife Management, 81, 1513–1519.

[ece310909-bib-0068] Kučas, A. , & Balčiauskas, L. (2020). Temporal patterns of ungulate‐vehicle collisions in Lithuania. Journal of Environmental Management, 273, 111172. 32768765 10.1016/j.jenvman.2020.111172

[ece310909-bib-0069] Kuznetsova, A. , Brockhoff, P. B. , & Christensen, R. H. B. (2017). lmerTest package: Tests in linear mixed effects models. Journal of Statistical Software, 82, 1–26.

[ece310909-bib-0070] Labonté, J. , Courtois, R. , & Ouellet, J.‐P. (1993). Déplacements et taille des domaines vitaux des orignaux (Alces alces) dans le Bas‐Saint‐Laurent et la Gaspésie. Ministère du Loisir, de la Chasse et de la Pêche. [French].

[ece310909-bib-0071] Lagos, L. , Picos, J. , & Valero, E. (2012). Temporal pattern of wild ungulate‐related traffic accidents in northwest Spain. European Journal of Wildlife Research, 58, 661–668.

[ece310909-bib-0072] Laliberté, J. , & St‐Laurent, M.‐H. (2020). In the wrong place at the wrong time: Moose and deer movement patterns influence wildlife‐vehicle collision risk. Accident Analysis & Prevention, 135, 105365. 31775075 10.1016/j.aap.2019.105365

[ece310909-bib-0073] Larsen, K. W. , & Boutin, S. (1994). Movements, survival, and settlement of red squirrel (*Tamiasciurus hudsonicus*) offspring. Ecology, 75, 214–223.

[ece310909-bib-0074] Laurian, C. , Dussault, C. , Ouellet, J.‐P. , Courtois, R. , & Poulin, M. (2012). Interactions between a large herbivore and a road network. Ecoscience, 19, 69–79.

[ece310909-bib-0075] Laurian, C. , Dussault, C. , Ouellet, J.‐P. , Courtois, R. , Poulin, M. , & Breton, L. (2008a). Behavioral adaptations of moose to roadside salt pools. Journal of Wildlife Management, 72, 1094–1100.

[ece310909-bib-0076] Laurian, C. , Dussault, C. , Ouellet, J.‐P. , Courtois, R. , Poulin, M. , & Breton, L. (2008b). Behavior of moose relative to a road network. Journal of Wildlife Management, 72, 1550–1557.

[ece310909-bib-0077] Lavsund, S. , & Sandegren, F. (1991). Moose‐vehicle relations in Sweden: A review. Alces, 27, 118–126.

[ece310909-bib-0078] Leblond, M. , Dussault, C. , & Ouellet, J.‐P. (2010). What drives fine‐scale movements of large herbivores? A case study using moose. Ecography, 33, 1102–1112.

[ece310909-bib-0079] Leblond, M. , Dussault, C. , & Ouellet, J.‐P. (2013). Avoidance of roads by large herbivores and its relation to disturbance intensity. Journal of Zoology, 289, 32–40.

[ece310909-bib-0080] Leblond, M. , Dussault, C. , Ouellet, J.‐P. , Poulin, M. , Courtois, R. , & Fortin, J. (2007). Management of roadside salt pools to reduce moose–vehicle collisions. Journal of Wildlife Management, 71, 2304–2310.

[ece310909-bib-0081] Leblond, M. , Frair, J. , Fortin, D. , Dussault, C. , Ouellet, J.‐P. , & Courtois, R. (2011). Assessing the influence of resource covariates at multiple spatial scales: An application to forest‐dwelling caribou faced with intensive human activity. Landscape Ecology, 26, 1433–1446.

[ece310909-bib-0082] Leclerc, M. , Dussault, C. , & St‐Laurent, M.‐H. (2012). Multiscale assessment of the impacts of roads and cutovers on calving site selection in woodland caribou. Forest Ecology and Management, 286, 59–65.

[ece310909-bib-0083] Lefort, S. , & Massé, S. (2015). Plan de gestion de l'orignal au Québec 2012–2019. Ministère des Forêts, de la Faune et des Parcs – Secteur de la faune et des parcs [French].

[ece310909-bib-0084] Lesmerises, F. , Déry, F. , Johnson, C. J. , & St‐Laurent, M.‐H. (2018). Spatiotemporal response of mountain caribou to the intensity of backcountry skiing. Biological Conservation, 217, 149–156.

[ece310909-bib-0085] Lesmerises, F. , Dussault, C. , & St‐Laurent, M.‐H. (2012). Wolf habitat selection is shaped by human activities in a highly managed boreal forest. Forest Ecology and Management, 276, 125–131.

[ece310909-bib-0086] Lesmerises, F. , Dussault, C. , & St‐Laurent, M.‐H. (2013). Major roadwork impacts the space use behaviour of gray wolf. Landscape and Urban Planning, 112, 18–25.

[ece310909-bib-0087] Lesmerises, R. , Rebouillat, L. , Dussault, C. , & St‐Laurent, M.‐H. (2015). Linking GPS telemetry surveys and scat analyses helps explain variability in black bear foraging strategies. PLoS One, 10, e0129857. 26132204 10.1371/journal.pone.0129857 PMC4489386

[ece310909-bib-0088] Liedvogel, M. , Chapman, B. B. , Muheimet, R. , & Åkesson, S. (2013). The behavioural ecology of animal movement: Reflections upon potential synergies. Animal Migration, 1, 39–46.

[ece310909-bib-0089] Loosen, A. E. , Devineau, O. , Zimmermann, B. , Cromsigt, J. P. , Pfeffer, S. E. , Skarpe, C. , & Mathisen, K. M. (2021). Roads, forestry, and wolves interact to drive moose browsing behavior in Scandinavia. Ecosphere, 12, e03358.

[ece310909-bib-0090] Lykkja, O. N. , Solberg, E. J. , Herfindal, I. , Wright, J. , Rolandsen, C. M. , & Hanssen, M. G. (2009). The effects of human activity on summer habitat use by moose. Alces, 45, 109–124.

[ece310909-bib-0091] Makin, D. F. , Chamaillé‐Jammes, S. , & Shrader, A. M. (2019). Alarm calls or predator calls: Which elicit stronger responses in ungulate communities living with and without lions? Oecologia, 190, 25–35. 30919106 10.1007/s00442-019-04391-3

[ece310909-bib-0092] Manly, B. F. J. , MacDonald, L. L. , Thomas, D. L. , MacDonald, T. L. , & Erickson, W. P. (2002). Resource selection by animals: Statistical analysis and design for field studies. Kluwer Academic Publishers.

[ece310909-bib-0093] Mata, C. , Ruiz‐Capillas, P. , & Malo, J. E. (2017). Small‐scale alterations in carnivore activity patterns close to motorways. European Journal of Wildlife Research, 63, 1–12.

[ece310909-bib-0094] Melin, M. , Matala, J. , Mehtätalo, L. , Pusenius, J. , & Packalen, T. (2023). The effect of snow depth on movement rates of GPS‐collared moose. European Journal of Wildlife Research, 69, 21.

[ece310909-bib-0095] Melin, M. , Matala, J. , Pusenius, J. , & Packalen, T. (2019). Calving and post‐calving habitat use of female moose in two contrasting landscapes. Wildlife Biology, 2019, 1–12.

[ece310909-bib-0096] Merkle, J. A. , Monteith, K. L. , Aikens, E. O. , Hayes, M. M. , Hersey, K. R. , Middleton, A. D. , Oates, B. A. , Sawyer, H. , Scurlock, B. M. , & Kauffman, M. J. (2016). Large herbivores surf waves of green‐up during spring. Proceedings of the Royal Society B, 283, 20160456. 27335416 10.1098/rspb.2016.0456 PMC4936031

[ece310909-bib-0097] Merrow, M. , Spoelstra, K. , & Roenneberg, T. (2005). The circadian cycle: Daily rhythms from behaviour to genes. EMBO Reports, 6, 930–935. 16222241 10.1038/sj.embor.7400541 PMC1369194

[ece310909-bib-0098] Miller, B. K. , & Litvaitis, J. A. (1992). Use of roadside salt licks by moose, *Alces alces*, in northern New Hampshire. Canadian Field‐Naturalist, 106, 112–117.

[ece310909-bib-0099] Ministère des Forêts, de la Faune et des Parcs (MFFP) . (2016). Inventaire aérien de la grande faune: Projet de la 85/185. Gouvernement du Québec. [French].

[ece310909-bib-0100] Mohr, C. O. (1947). Table of equivalent populations of north American small mammals. American Midland Naturalist, 37, 223–249.

[ece310909-bib-0101] Mosnier, A. , Ouellet, J.‐P. , & Courtois, R. (2008). Black bear adaptation to low productivity in the boreal forest. Ecoscience, 15, 485–497.

[ece310909-bib-0102] Muhly, T. B. , Johnson, C.‐A. , Hebblewhite, M. , Neilson, E. W. , Fortin, D. , Fryxell, J. M. , Latham, A. D. M. , Latham, M. C. , McLoughlin, P. D. , Merrill, E. , Paquet, P. C. , Patterson, B. R. , Schmiegelow, F. , Scurrah, F. , & Musiani, M. (2019). Functional response of wolves to human development across boreal North America. Ecology and Evolution, 9, 10801–10815. 31624583 10.1002/ece3.5600 PMC6787805

[ece310909-bib-0103] Mumma, M. A. , Gillingham, M. P. , Marshall, S. , Procter, C. , Bevington, A. R. , & Scheideman, M. (2021). Regional moose (*Alces alces*) responses to forestry cutblocks are driven by landscape‐scale patterns of vegetation composition and regrowth. Forest Ecology and Management, 481, 118763.

[ece310909-bib-0104] Nathan, R. , Getz, W. M. , Revilla, E. , Holyoak, M. , Kadmon, R. , Saltz, D. , & Smouse, P. E. (2008). A movement ecology paradigm for unifying organismal movement research. Proceedings of the National Academy of Sciences, 105, 19052–19059. 10.1073/pnas.0800375105 PMC2614714 19060196

[ece310909-bib-0105] National Research Council Canada (NRC) . (2021). Sunrise/sunset calculator . https://www.nrc‐cnrc.gc.ca/eng/services/sunrise/advanced.html

[ece310909-bib-0106] Neumann, W. , & Ericsson, G. (2018). Influence of hunting on movements of moose near roads. Journal of Wildlife Management, 82, 918–928.

[ece310909-bib-0107] Parker, K. L. , Robbins, C. T. , & Hanley, T. A. (1984). Energy expenditures for locomotion by mule deer and elk. Journal of Wildlife Management, 48, 474–488.

[ece310909-bib-0108] Passoni, G. , Coulson, T. , Ranc, N. , Corradini, A. , Hewison, A. M. , Ciuti, S. , Gehr, B. , Heurich, M. , Brieger, F. , Sandfort, R. , Mysterud, A. , Balkenhol, N. , & Cagnacci, F. (2021). Roads constrain movement across behavioural processes in a partially migratory ungulate. Movement Ecology, 9, 1–12. 34774097 10.1186/s40462-021-00292-4 PMC8590235

[ece310909-bib-0109] Patterson, B. R. , & Messier, F. (2003). Age and condition of deer killed by coyotes in Nova Scotia. Canadian Journal of Zoology, 81, 1894–1898.

[ece310909-bib-0110] Pinard, V. , Dussault, C. , Ouellet, J.‐P. , Fortin, D. , & Courtois, R. (2012). Calving rate, calf survival rate, and habitat selection of forest‐dwelling caribou in a highly managed landscape. Journal of Wildlife Management, 76, 189–199.

[ece310909-bib-0111] Pokallus, J. W. , & Pauli, J. N. (2016). Predation shapes the movement of a well‐defended species, the north American porcupine, even when nutritionally stressed. Behavioral Ecology, 27, 470–475.

[ece310909-bib-0112] Polfus, J. L. , Hebblewhite, M. , & Heinemeyer, K. (2011). Identifying indirect habitat loss and avoidance of human infrastructure by northern mountain woodland caribou. Biological Conservation, 144, 2637–2646.

[ece310909-bib-0113] Quinn, G. P. , & Keough, M. J. (2002). Experimental design and data analysis for biologists. Cambridge University Press.

[ece310909-bib-0114] R Core Team . (2021). R: A language and environment for statistical computing. R Foundation for Statistical Computing.

[ece310909-bib-0115] Rea, R. V. , Scheideman, M. C. , Hesse, G. , & Mumma, M. A. (2021). The effectiveness of decommissioning roadside mineral licks on reducing moose (*Alces alces*) activity near highways: Implications for moose–vehicle collisions. Canadian Journal of Zoology, 99, 1009–1019.

[ece310909-bib-0116] Richard, Q. , Toïgo, C. , Appolinaire, J. , Loison, A. , & Garel, M. (2017). From gestation to weaning: Combining robust design and multi‐event models unveils cost of lactation in a large herbivore. Journal of Animal Ecology, 86, 1497–1509. 28772345 10.1111/1365-2656.12736

[ece310909-bib-0117] Robb, B. S. , Merkle, J. A. , Sawyer, H. , Beck, J. L. , & Kauffman, M. J. (2022). Nowhere to run: Semi‐permeable barriers affect pronghorn space use. Journal of Wildlife Management, 86, e22212.

[ece310909-bib-0118] Robertson, E. P. , Fletcher, R. J. , Cattau, C. E. , Udell, B. J. , Reichert, B. E. , Austin, J. D. , & Valle, D. (2018). Isolating the roles of movement and reproduction on effective connectivity alters conservation priorities for an endangered bird. Proceedings of the National Academy of Sciences, 115, 8591–8596. 10.1073/pnas.1800183115 PMC6112689 30082379

[ece310909-bib-0119] Robitaille, A. , & Saucier, J.‐P. (1998). Paysages régionaux du Québec méridional. Les publications du Québec [French].

[ece310909-bib-0120] Rochette, B. , & Dumont, J.‐F. (2022). Inventaire aérien de l'orignal dans la réserve faunique des Laurentides à l'hiver 2020. Ministère des Forêts, de la Faune et des Parcs, Québec [French].

[ece310909-bib-0121] Rubin, M. J. , Brock, M. T. , Davis, A. M. , German, Z. M. , Knapp, M. , Welch, S. M. , Harmer, S. L. , Maloof, J. N. , Davis, S. J. , & Weinig, C. (2017). Circadian rhythms vary over the growing season and correlate with fitness components. Molecular Ecology, 26, 5528–5540. 28792639 10.1111/mec.14287

[ece310909-bib-0122] Rudolph, T. D. , & Drapeau, P. (2012). Using movement behaviour to define biological seasons for woodland caribou. Rangifer, 20, 295–307.

[ece310909-bib-0123] Rueda, M. , Rebollo, S. , Gálvez‐Bravo, L. , & Escudero, A. (2008). Habitat use by large and small herbivores in a fluctuating Mediterranean ecosystem: Implications of seasonal changes. Journal of Arid Environments, 72, 1698–1708.

[ece310909-bib-0124] Severud, W. J. , DelGiudice, G. D. , & Obermoller, T. R. (2019). Association of moose parturition and post‐parturition habitat with calf survival. Journal of Wildlife Management, 83, 175–183.

[ece310909-bib-0125] Shepard, D. B. , Kuhns, A. R. , Dreslik, M. J. , & Phillips, C. A. (2008). Roads as barriers to animal movement in fragmented landscapes. Animal Conservation, 11, 288–296.

[ece310909-bib-0126] Spoelstra, K. , Albrecht, U. , Van der Horst, G. T. , Brauer, V. , & Daan, S. (2004). Phase responses to light pulses in mice lacking functional per or cry genes. Journal of Biological Rhythms, 19, 518–529. 15523113 10.1177/0748730404268122

[ece310909-bib-0127] Stewart, D. G. , Gulsby, W. D. , Ditchkoff, S. S. , & Collier, B. A. (2022). Spatiotemporal patterns of male and female white‐tailed deer on a hunted landscape. Ecology and Evolution, 12, e9277. 36110880 10.1002/ece3.9277 PMC9465197

[ece310909-bib-0128] St‐Pierre, F. , Drapeau, P. , & St‐Laurent, M.‐H. (2022). Stairway to heaven or highway to hell? How characteristics of forest roads shape their use by large mammals in the boreal forest. Forest Ecology and Management, 510, 120108.

[ece310909-bib-0129] Street, G. M. , Rodgers, A. R. , & Fryxell, J. M. (2015). Mid‐day temperature variation influences seasonal habitat selection by moose. Journal of Wildlife Management, 79, 505–512.

[ece310909-bib-0130] Thompson, D. G. , & Pitt, D. G. (2003). A review of Canadian forest vegetation management research and practice. Annals of Forest Science, 60, 559–572.

[ece310909-bib-0131] Thornton, D. H. , Sunquist, M. E. , & Main, M. B. (2004). Ecological separation within newly sympatric populations of coyotes and bobcats in south‐central Florida. Journal of Mammalogy, 85, 973–982.

[ece310909-bib-0132] Tipton, E. , Hallberg, K. , Hedges, L. V. , & Chan, W. (2017). Implications of small samples for generalization: Adjustments and rules of thumb. Evaluation Review, 41, 472–505. 27402612 10.1177/0193841X16655665

[ece310909-bib-0133] Trombulak, S. C. , & Frissell, C. A. (2000). Review of ecological effects of roads on terrestrial and aquatic communities. Conservation Biology, 14, 18–30.

[ece310909-bib-0134] Van Ballenberghe, V. , & Ballard, W. B. (2007). Population dynamics. In C. C. Schwartz , A. E. Franzmann , & R. E. McCabe (Eds.), Ecology and Management of the North American Moose (2nd ed., pp. 223–245). University Press of Colorado.

[ece310909-bib-0135] Van Langevelde, F. , Van Dooremalen, C. , & Jaarsma, C. F. (2009). Traffic mortality and the role of minor roads. Journal of Environmental Management, 90, 660–667. 18079047 10.1016/j.jenvman.2007.09.003

[ece310909-bib-0136] Villemure, M. , & Jolicoeur, H. (2004). First confirmed occurrence of a wolf, *Canis lupus*, south of the St. Lawrence River in over 100 years. Canadian Field‐Naturalist, 118, 608–610.

[ece310909-bib-0137] Vistnes, I. I. , Nellemann, C. , Jordhøy, P. , & Støen, O. G. (2008). Summer distribution of wild reindeer in relation to human activity and insect stress. Polar Biology, 31, 1307–1317.

[ece310909-bib-0138] Wattles, D. W. , Zeller, K. A. , & DeStefano, S. (2018). Response of moose to a high‐density road network. Journal of Wildlife Management, 82, 929–939.

[ece310909-bib-0139] Whittington, J. , Hebblewhite, M. , DeCesare, N. J. , Neufled, L. , Bradley, M. , Wilmhurst, J. , & Musiani, M. (2011). Caribou encounters with wolves increase near roads and trails: A time‐to‐event approach. Journal of Applied Ecology, 48, 1535–1542.

[ece310909-bib-0140] Wronski, T. , Apio, A. , Baranga, J. , & Plath, M. (2006). Scent marking and territorial defence in male bushbuck (*Tragelaphus scriptus*). Journal of Zoology, 270, 49–56.

[ece310909-bib-0141] Young, M. E. , Ryberg, W. A. , Fitzgerald, L. A. , & Hibbitts, T. J. (2018). Fragmentation alters home range and movements of the dunes sagebrush lizard (*Sceloporus arenicolus*). Canadian Journal of Zoology, 96, 905–912.

[ece310909-bib-0142] Zuur, A. , Ieno, E. N. , & Smith, G. M. (2007). Analyzing ecological data. Springer Science & Business Media.

