# Supplementary material for: Temporal variations in female moose responses to roads and logging in the absence of wolves
Source: Ecol Evol. 2024 Feb 1;14(2):e10909. doi: 10.1002/ece3.10909 (PMC10834149; doi:10.1002/ece3.10909)
Supplement: Supplementary file 1 — Appendix S1 [file ECE3-14-e10909-s001.docx]

**SUPPORTING INFORMATION**

Gagnon, M., F. Lesmerises and M.-H. St-Laurent. Temporal variations in female moose responses to roads and logging in the absence of wolves. Ecology and Evolution.

**Table S1.** Links between dependent variables, hypotheses, predictions, and geomatics and statistical analyses used to describe the habitat selection and space use behaviors of female moose (*n* = 18) collared with GPS devices near the Claude-Béchard Highway in the Bas-Saint-Laurent region, Canada, in 2017.

| **Dependent variables** | **Hypotheses** | **Predictions** | **Geomatics** | **Statistical analyses** |
| --- | --- | --- | --- | --- |
| Home-range size | Movement costs | 1) smaller in winter | Kernel Brownian bridges >95% | ANOVA + Tukey |
| Home-range composition | Movement costs | 2) larger when there is a greater proportion of shelter habitat | Kernel Brownian bridges >95% | Mixed regression (model selection based on AIC_c_) |
| Movement rates | Resource acquisition, movement costs | 3) greater in spring/green-up, summer/rearing and fall/rut | Ellipses >75% Brownian bridges | ANOVA + Tukey |
|  | Risk mitigation | 4) greater at dusk-night-dawn | Ellipses >75% Brownian bridges | Mixed regression  (model selection based on AIC_c_) |
|  | Resource acquisition, risk mitigation | 5) greater near paved and forest roads | Ellipses >75% Brownian bridges | Mixed regression  (model selection based on AIC_c_) |
| Habitat selection | Resource acquisition, movement costs, risk mitigation | 6) food habitat at dusk-night-dawn during spring/green-up, summer/rearing and fall/rut | MCP 100% (use/ availability) | Mixed logistic regression  (model selection based on AIC_c_) |
|  | Resource acquisition, risk mitigation | 7) paved and forest roads at dusk-night-dawn during spring/green-up, summer/rearing and fall/rut | MCP 100% (use/ availability) | Mixed logistic regression  (model selection based on AIC_c_) |
|  | Risk mitigation | 8) shelter habitat during calving | MCP 100% (use/ availability) | Mixed logistic regression  (model selection based on AIC_c_) |

**Table S2.** Average cut-off dates for each biological period per year (± SD) of female moose (*n* = 18) captured in 2017 and monitored until May 2019 near the Claude-Béchard Highway in the Bas-Saint-Laurent region, Canada. Breaks were identified using the distribution of mean movement rates in function of Julian days for each individual-year combination.

| **Biological periods** | **Average dates ± SD** | | |
| --- | --- | --- | --- |
|  | **2017** | **2018** | **2019** |
| Spring/green-up | April 27^th^ ± 5 | April 28^th^ ± 5 | April 25^th^ ± 4 |
| Calving | May 20^th^ ± 8 | May 19^th^ ± 3 | – |
| Summer/rearing | June 5^th^ ± 9 | June 8^th^ ± 7 | – |
| Fall/rut | October 6^th^ ± 11 | September 29^th^ ± 3 | – |
| Winter | December 2^nd^ ± 16 | November 23^th^ ± 11 | – |

**Table S3.** Candidate models, ΔAIC_c_ and the number of parameters (*k*)^1^ for the linear mixed regression used to explain variations in movement rates of female moose (n = 18) captured in 2017 near the Claude-Béchard Highway in the Bas-Saint-Laurent region, Canada (*n* _(ID-year)_ = 43 winter, 41 spring/green-up, 32 calving, 32 summer/rearing, 27 fall/rut). The most parsimonious model is shown in bold for each biological period.

| **Models^2^** | **Winter** | | **Spring/ green-up** | | **Calving** | | **Summer/ rearing** | | | **Fall/rut** | |
| --- | --- | --- | --- | --- | --- | --- | --- | --- | --- | --- | --- |
|  | **ΔAIC_c_** | ***k*** | **ΔAIC_c_** | ***k*** | **ΔAIC_c_** | ***k*** | **ΔAIC_c_** | ***k*** | **ΔAIC_c_** | | ***k*** |
| Topography | 373.90 | 3 | 72.09 | 3 | 152.37 | 1 | 2061.49 | 2 | 311.11 | | 1 |
| Topography + Day phase | 172.64 | 4 | 58.90 | 4 | 29.88 | 2 | 148.97 | 3 | 25.22 | | 2 |
| Topography + Day phase + Paved road + Day phase*Paved road | 161.21 | 6 | 52.94 | 6 | 32.25 | 4 | 100.16 | 5 | 21.77 | | 4 |
| **Topography + Day phase + Forest road + Day phase*Forest road** | 104.00 | 6 | 6.90 | 6 | **0.00** | **4** | 62.88 | 5 | 31.08 | | 4 |
| **Topography + Day phase + Paved road + Forest road + Day phase*Paved road + Day phase*Forest road** | 84.67 | 8 | **0.00** | **8** | 2.05 | 6 | 12.64 | 7 | 27.40 | | 6 |
| Topography + Day phase + Proportion of 4-7 m deciduous and mixed + Proportion of 0-4 m habitats + Day phase*Proportion of 4-7 m deciduous and mixed + Day phase*Proportion of 0-4 m habitats | 97.59 | 8 | 61.61 | 8 | 40.79 | 6 | 122.86 | 70 | 11.33 | | 6 |
| **Topography + Day phase + Paved road + Forest road + Proportion of 0-4** **m habitats + Proportion of 4-7** **m deciduous and mixed** | **0.00** | **8** | 0.43 | 8 | 7.85 | 6 | **0.00** | **7** | **0.00** | | **6** |

^1^ The number of parameters did not include ID-year as a random factor.

^2^ The topographic variables changed depending on the biological period (see *Statistical Analyses*). Also, see section *2.5 – Statistical analyses* for the paved and forest road buffer zone widths used in the different biological periods.

**Table S4.** Candidate models, ΔAIC_c_, and number of parameters (*k*) for the linear mixed regression used to explain home-range size of female moose (*n* = 18) captured in 2017 near the Claude-Béchard Highway in the Bas-Saint-Laurent region, Canada (*n* _(ID)_ = 18 in winter, spring/green-up, calving and summer/rearing, 14 in fall/rut). The most parsimonious model is shown in bold for each biological period.

| **Models^1^** | ***k*^2^** | **ΔAIC_c_** | | | | |
| --- | --- | --- | --- | --- | --- | --- |
|  |  | **Winter** | **Spring/ green-up** | **Calving** | **Summer/rearing** | **Fall/rut** |
| Elevation | 1 | 17.72 | 7.39 | 17.26 | 7.47 | 4.07 |
| Elevation + Proportion of shelter habitat | 3 | 16.46 | 1.57 | 15.24 | 1.28 | 1.85 |
| Elevation + Proportion of food habitat | 3 | 4.89 | 5.84 | 12.03 | 1.93 | 0.55 |
| **Elevation + Proportion of shelter habitat + Proportion of food habitat** | 5 | 5.00 | **0.00** | 11.58 | **0.00** | **0.00** |
| Elevation + Proportion of shelter habitat + Forest road density | 4 | 11.38 | 6.22 | 3.46 | 5.73 | 6.73 |
| **Elevation + Proportion of food habitat + Forest road density** | 4 | 1.26 | 10.35 | **0.00** | 5.72 | 3.64 |
| Elevation + Proportion of shelter habitat + Proportion of food habitat + Forest road density | 6 | 0.26 | 5.00 | 0.78 | 4.77 | 4.17 |
| Elevation + Proportion of shelter habitat + Road density | 4 | 10.54 | 6.18 | 11.76 | 5.33 | 6.34 |
| Elevation + Proportion of food habitat + Road density | 4 | 1.15 | 10.56 | 8.51 | 5.16 | 3.22 |
| **Elevation + Proportion of shelter habitat + Proportion of food habitat + Road density** | 6 | **0.00** | 5.03 | 8.84 | 3.58 | 3.62 |

^1^ Shelter habitat included the proportion of both low- and high-density coniferous stands +7 m. Food habitat included the proportion of 0-4 m habitats and 4-7 m deciduous and mixed stands. Road density included all roads (paved and forest roads).

^2^ The number of parameters did not include ID as a random factor.

**Table S5.** Candidate models, ΔAICc and the number of parameters (*k*)^1^ for the mixed logistic regression used to assess the habitat selection of female moose (n = 18) captured in 2017 near the Claude-Béchard Highway in the Bas-Saint-Laurent region, Canada (*n* _(ID-year)_ = 43 winter, 41 spring/green-up, 32 calving, 32 summer/rearing, 27 fall/rut). The most parsimonious model is shown in bold.

| **Models^2^** | **Winter** | | **Spring/ green-up** | | **Calving** | | **Summer/ rearing** | | **Fall/rut** | |
| --- | --- | --- | --- | --- | --- | --- | --- | --- | --- | --- |
|  | **ΔAIC_c_** | ***k*** | **ΔAIC_c_** | ***k*** | **ΔAIC_c_** | ***k*** | **ΔAIC_c_** | ***k*** | **ΔAIC_c_** | ***k*** |
| Landcover type + Topography | 1344.45 | 9 | 377.78 | 8 | 324.06 | 8 | 2736.79 | 9 | 612.15 | 9 |
| Model 1 + Day phase + Landcover type^3^*Day phase | 1289.46 | 14 | 304.44 | 13 | 296.39 | 13 | 1584.43 | 14 | 374.01 | 14 |
| Model 1 + Day phase + Paved road + Forest road | 89.48 | 12 | 103.72 | 11 | 20.21 | 11 | 421.03 | 12 | 197.44 | 12 |
| **Model 1 + Day phase + Paved road + Forest road + Paved road*Day phase + Forest road*Day phase** | **0.00** | **14** | **0.00** | **13** | **0.00** | **13** | **0.00** | **14** | **0.00** | **14** |
| Model 1 + Day phase + Forest road | 195.10 | 11 | 330.73 | 10 | 89.24 | 10 | 1729.27 | 11 | 482.10 | 11 |
| Model 1 + Day phase + Forest road + Forest road*Day phase | 102.83 | 12 | 235.81 | 11 | 70.00 | 11 | 1363.21 | 12 | 331.56 | 12 |
| Model 1 + Paved road + Forest road | 89.66 | 11 | 101.91 | 10 | 18.34 | 10 | 420.10 | 11 | 201.42 | 11 |
| Model 1 + Paved road | 1258.92 | 10 | 166.34 | 9 | 263.20 | 9 | 1448.75 | 10 | 333.12 | 10 |
| Model 1 + Forest road | 195.11 | 10 | 329.05 | 9 | 87.48 | 9 | 1728.34 | 10 | 488.01 | 10 |

^1^ The number of parameters did not include ID-year as a random factor.

^2^ The topographic variables changed depending on the biological period (see *Statistical Analyses*). Also, see section *2.5 - Statistical analyses* for the paved and forest road buffer zone widths used in the different biological periods.

^3^ Only these landcover types were used in interaction with day phases: 0-4 m (height) habitats, 4-7 m deciduous and mixed stands, +7 m low-density coniferous stands, +7 m high-density coniferous stands.
